# Supplementary material for: Generation of “Off-the-Shelf” Natural Killer Cells from Peripheral Blood Cell-Derived Induced Pluripotent Stem Cells
Source: Stem Cell Reports. 2017 Nov 22;9(6):1796–812. doi: 10.1016/j.stemcr.2017.10.020 (PMC5785702; doi:10.1016/j.stemcr.2017.10.020)
Supplement: Document S1. Supplemental Experimental Procedures, Figures S1 and S2, and Tables S1–S3 [file mmc1.pdf]

Stem Cell Reports, Volume *9*

Supplemental Information

Generation of "Off-the-Shelf" Natural Killer Cells from Peripheral Blood Cell-Derived Induced Pluripotent Stem Cells

Jieming Zeng, Shin Yi Tang, Lai Ling Toh, and Shu Wang

Supplemental Information

Generation of "Off-the-Shelf" Natural Killer Cells from Peripheral Blood Cell-derived Induced Pluripotent Stem Cells

Jieming Zeng[1], Shin Yi Tang[1,2], Lai Ling Toh[1] and Shu Wang[1,2]

[1]Institute of Bioengineering and Nanotechnology, Singapore
[2]Department of Biological Sciences, National University of Singapore, Singapore

Inventory of Supplemental Information

1. Supplemental Data

Figure S1, related to Figure 3c;

Figure S2, related to Figure 7;

Table S1, related to Figure 3;

Table S2, related to Figure 7;

Table S3, related to discussion.

2. Supplemental Experimental Procedures

3. References

Supplemental Experimental Procedures

Cell culture. A hESC line, H1 (WiCell Research Institute, Madison, WI, http://www.wicell.org) and a previously generated fibroblast-derived iPSC line, iPSC#5.9[1], were cultured with mTeSR1 (StemCell Technologies, Vancouver, BC, Canada, http://www.stemcell.com) on Matrigel (BD Biosciences, San Diego, CA, http://www.bdbiosciences.com) -coated six-well plates. Cell lines: OP9, K562, Raji, SK-OV-3, SW480, HCT-8, MCF-7 and SCC-25 (American Type Culture Collection [ATTC], Manassas, VA, http://www.atcc.org) were cultured as recommended by ATCC. Cell line OP9-DLL1 (Riken BRC Cell Bank, Ibaraki, Japan, http://cell.brc.riken.jp/en/) was cultured in α-MEM (Thermo Fisher Scientific, Waltham, MA, http://corporate.thermofisher.com) supplemented with 20% fetal bovine serum (FBS) (HyClone, Logan, UT, http://www.hyclone.com). Cell line K562-mbIL15-41BBL (kindly provided by Dr. D. Campana, Yong Loo Lin School of Medicine, National University of Singapore) was cultured in IMDM (Thermo Fisher Scientific) supplemented with 10% FBS.

Generation of PBC-iPSCs. To generate iPSCs from PBCs, frozen PBMCs from a healthy donor (StemCell Technologies) were thawed and cultured with 5 μg/ml phytohemagglutinin (PHA; Sigma-Aldrich, St Louis, MO, http://www.sigmaaldrich.com) for 2 days in complete RPMI 1640 medium, which is composed of RPMI 1640, 10% heat-inactivated human serum AB (Gemini Bio-Products, West Sacramento, CA, http://www.gembio.com), 2 mM L-glutamine (Thermo Fisher Scientific) and 0.1 mM nonessential amino acids (Thermo Fisher Scientific). The cultured blood cells were then transduced with Sendai reprogramming vectors from a CytoTune iPS 2.0 Sendai Reprogramming Kit (Thermo Fisher Scientific) at MOI of 5:5:3 (KOS, hc-Myc, hKlf4) in complete RPMI 1640 medium containing 10 ng/ml of IL-2, IL-7 and IL-15 overnight. The transduced cells were then washed and cultured for 5 days before seeding to a six-well plate grown with mitomycin C (Sigma-Aldrich) -treated mouse embryonic fibroblasts (mEFs). Half of medium was replaced on day 1 to 3 after seeding with iPSC medium, which is composed of DMEM/F12 (Thermo Fisher Scientific), 20% knockout serum replacement (Thermo Fisher Scientific), 2 mM L-glutamine, 1% nonessential amino acids, 0.1 mM 2-mercaptoethanol and 5 ng/ml basic fibroblast growth factor (PeproTech). Three to four weeks after seeding, the resulting iPSCs were first expanded on mEFs in iPSC medium and later on Matrigel-coated plates in mTeSR1.

To generate non-T cell-derived PBC-iPSCs, PBMCs from healthy donors were cultured in CD34+ cells enrichment medium, which is composed of StemPro-34 medium (Thermo Fisher Scientific), 100 ng/ml SCF, 50 ng/ml IL-3 (PeproTech) and 25 ng/ml GM-CSF (PeproTech) for three days. At day 0, episomal reprogramming vectors from a Epi5 Episomal iPSC Reprogramming Kit (Thermo Fisher Scientific) were delivered into the cultured PBMCs via nucleofection using a Amaxa Nucleofector 2b (Lonza, http://www.lonza.com). The nuleofected cells were then seeded on mitomycin C-inactivated mEFs in CD34+ cell enrichment medium. On day 2, the cells were adapted to a 1:1 mixture of CD34+ cells enrichment medium: iPSC medium. From day 3 on, the cells were cultured in iPSC medium, which was changed every other day. Two to four weeks after seeding, iPSC colonies were picked and expanded in Matrigel-coated six-well plates in mTeSR1.

TCRB and TCRG gene clonality assays. Genomic DNA was isolated from PBC-iPSCs using a DNeasy Blood and Tissue Kit (Qiagen, https://www.qiagen.com) according to the manufacturer's instruction. To detect TCRβ and TCRγ gene rearrangement in genomic DNA, PCR was carried out with master mixes provided in TCRB and TCRG Gene Clonality Assay kits (Invivoscribe Technologies, San Diego, CA, http://www.invivoscribe.com) and AmpliTaq Gold DNA polymerase (Thermo Fisher Scientific) using the following program: 95ºC for 7 minutes; 35 amplification cycles (95ºC for 45 seconds, 60ºC for 45 seconds, 72ºC for 90 seconds); and final extension of 72ºC for 10 minutes before holding at 15ºC. PCR products were separated by electrophoresis in 2% MetaPhor Agarose (Lonza, http://www.lonza.com) gel.

Flow cytometry. To study phenotypic change during hPSC differentiation, cells were harvested and stained using antibodies against CD34 (BD Biosciences, cat#550761 and cat#555824), CD43 (BD Biosciences, cat#555475), CD45 (BD Biosciences, cat#555483 and cat#557748), CD56 (BD Biosciences, cat#555518), TCRαβ (BD Biosciences, cat#564728), CD3 (BD Biosciences, cat#555333 and cat#561811), CD4 (BD Biosciences, cat#557852), CD8 (BD Biosciences, cat#555634), CD19 (BD Biosciences, cat#561741), CD14 (Thermo Fisher Scientific, cat#12-0149-42), NKp30 (BD Biosciences, cat#558407), NKp44 (BD Biosciences, cat#558563), NKp46 (BD Biosciences, cat#557991), NKG2D (BD Biosciences, cat#557940), NKG2A (Beckman Coulter, cat#IM3291U), CD94 (Thermo Fisher Scientific, cat#12-0949-42), CD16 (BD Biosciences, cat#560995), FasL (BD Biosciences, cat#564261), TRAIL (BD Biosciences, cat#561784), DNAM-1 (BD Biosciences, cat#559789), CD158a,h (KIR2DL1/S1) (Beckman Coulter, cat#A09778), CD158b (KIR2DL2/L3/S2) (Miltenyi Biotec, cat#130-092-618), CD158f (KIR2DL5) (Miltenyi Biotec, cat#130-096-199), CD158i (KIR2DS4) (Miltenyi Biotec, cat#130-092-680), CD158e1/e2 (KIR3DL1/S1) (Beckman Coulter,

cat#IM3292) and CD158e/k (KIR3DL1/L2) (Miltenyi Biotec, cat#130-095-205) and analyzed with a FACSCalibur flow cytometer (BD Biosciences). Isotype controls were used accordingly for flow cytometry.

ELISPOT assay. To detect IFN-γ secretion, a Human IFN-γ ELISpotPro kit (MABTECH, Nacka Strand, Sweden, https://www.mabtech.com) was used. In brief, 0 to 10×10^4 PBC-iPSC-NK cells and 5×10^4 K562 or Raji cells were cocultured on a IFN-γ ELISPOT plate overnight. IFN-γ spots were stained according to the manufacturer's manual. To measure GrB secretion, a Human Granzyme B ELISpot Kit (R&D Systems, Minneapolis, MN, https://www.rndsystems.com) was used. In brief, 0 to 1.2×10^4 PBC-iPSC-NK cells were incubated with or without 5×10^4 K562 cells on a human GrB microplate for 4 hours. GrB spots were then stained as described in the manufacturer's manual. IFN-γ and GrB spots were counted using an ImmunoSpot Analyzer (CTL, Shaker Heights, OH, http://www.immunospot.com).

Cytotoxicity and ADCC assay. To detect direct cytotoxicity of PBC-iPSC-NK cells against target cells, a flow cytometry-based method was used. In brief, 0 to 0.5×10^6 PBC-iPSC-NK cells were cocultured with 2×10^4 carboxyfluorescein diacetate succinimidyl ester (CFSE; Thermo Fisher Scientific) -labelled cancer cells at various effector to target (E:T) ratios for 4-6 hours. Samples were then stained on ice with 7-Amino-Actinomycin D (7-AAD, BD Biosciences) for 10 minutes. After washing, target cell death was assessed with flow cytometer by the percentage of 7-AAD-stained cells in CFSE-positive population. To evaluate ADCC function of PBC-iPSC-NK cells, cocultures of NK cells and CFSE-labelled Raji cells were set up at the indicated E:T ratios in the presence of human IgG1 (Sigma-Aldrich), anti-CD20-mIgG1 (InvivoGen, San Diego, CA, http://www.invivogen.com) or anti-CD20-hIgG1 (InvivoGen) of various concentrations, Raji cell death was measured after 4-hour incubation by flow cytometry as described above.

Expansion of fresh and cryopreserved PBC-iPSC-NK cells. To expand fresh PBC-iPSC-NK cells, 0.25-1×10^6 purified NK cells were cocultured with γ- irradiated (100 Gy) K562-mbIL15-41BBL cells at an NK cell: feeder cell ratio of 1:10 in gas-permeable G-Rex10 flasks (Wilson Wolf Manufacturing, New Brighton, MN, http://www.wilsonwolf.com) in 40 ml CellGro SCGM serum-free medium (Cell Genix, Freiburg, Germany, http://www.cellgenix.com) supplemented with 10% FBS, 20 IU/ml IL-2 (PeproTech), 10 ng/ml SCF, 5 ng/ml FLT3L, 5 ng/ml IL-7 and 10 ng/ml IL-15. Fresh cytokines were replenished every 2-3 days and 30 ml medium was replaced with fresh medium every 5 days during the 14-day expansion. Numbers of NK cells were counted at every medium change. To cryopreserve PBC-iPSC-NK cells, NK cells were resuspended in cold CryoStor CS10 (BioLife Solutions, Bothell, WA, http://www.biolifesolutions.com/) and frozen at -80°C in a Mr. Frosty (Thermo Fisher Scientific) overnight before storing in liquid nitrogen. To study the expansion of cryopreserved PBC-iPSC-NK cells, the NK cells were thawed and cell viability and live cell number were determined before and after coculture with K562-mbIL15-41BBL.

Expansion of donor-derived PB-NK cells. To derive PB-NK cells, 2×10^6 PBMCs from healthy donors were cocultured with 4×10^6 γ-irradiated (100 Gy) K562-mbIL15-41BBL cells in CellGro SCGM serum-free medium supplemented with 10% FBS and 50 IU/mL IL-2 using T75 flasks in upright position. Half of the medium was replaced with fresh medium and fresh IL-2 was replenished every 2-3 days until day 7. Hereafter, 2×10^6 cells were re-stimulated weekly with 2×10^6 K562-mbIL15-41BBL cells for another two weeks. The cells were harvested on day 21 for experiments.

Derivation of primary tumor cells. To test the cytotoxicity of NK cells against primary tumor cells, short-term cultures of primary tumor cells were established from fresh colorectal cancer samples using a protocol described previously[2]. The samples were obtained from patients under treatment at National Cancer Centre Singapore and the study was approved by local Institutional Review Board. In brief, the tumor samples were finely minced and further dissociated into very small tissue fragments by vigorous pipetting. The tissue fragments were then resuspended in AR-5 initiation medium [2] and grown in cell culture dishes to establish primary cultures. Upon heavy growth of tumor cells, the adherent colonies were scraped off under a stereomicroscope for further culture. Such derived pure cancer cells were then propagated in RPMI 1640 medium supplemented with 10% FBS. These cancer cells were characterized by immunostaining using antibodies against cytokeratin (Miltenyi Biotec, cat#130-080-101), vimentin (BD Biosciences, cat#562337), CD133 (BD Biosciences, cat#566593), CK20 (Santa Cruz, cat#sc-271183) and flow cytometry using antibodies against CD133, CD44 (Miltenyi Biotec, cat#130-095-177) and EpCAM (Miltenyi Biotec, cat#130-091-254) and then used as target cells in cytotoxicity assay between passage 10 to 12.

KIR typing. To detect KIR genes on genomic DNA level and to analyze of KIR expression on mRNA level, a KIR typing kit (Miltenyi Biotec) was used according to manufacturer's instruction.

References

1. Yang, J. et al. Tumor tropism of intravenously injected human-induced pluripotent stem cell-derived neural stem cells and their gene therapy application in a metastatic breast cancer model. *Stem cells* **30**, 1021-1029 (2012).

2. Park, J.G., Ku, J.L. & Park, S.Y. Isolation and culture of colon cancer cell lines. *Methods in molecular medicine* **88**, 79-92 (2004).

Figure S1

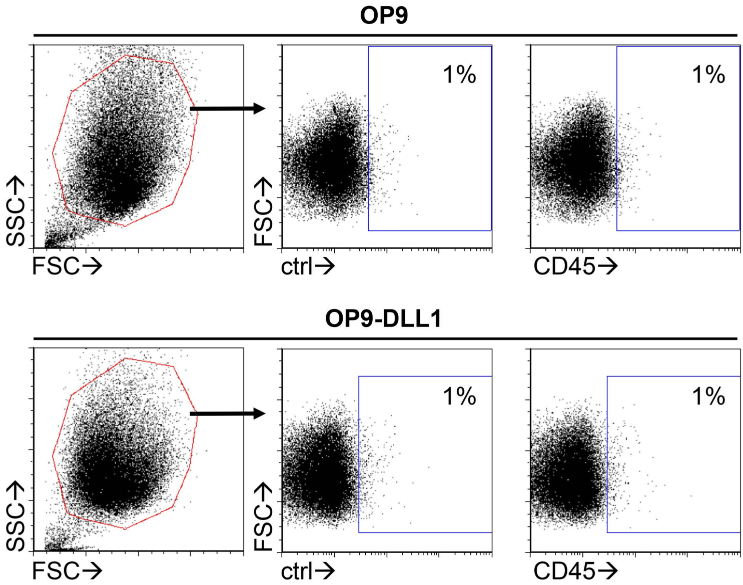

Fig. S1: CD45 expression in OP9 and OP9-DLL1. OP9 and OP9-DLL1 cells were stained by an anti-human CD45 monoclonal antibody and its isotype control. Flow cytometric analysis showed no CD45 expression in both OP9 and OP9-DLL1.

Figure S2

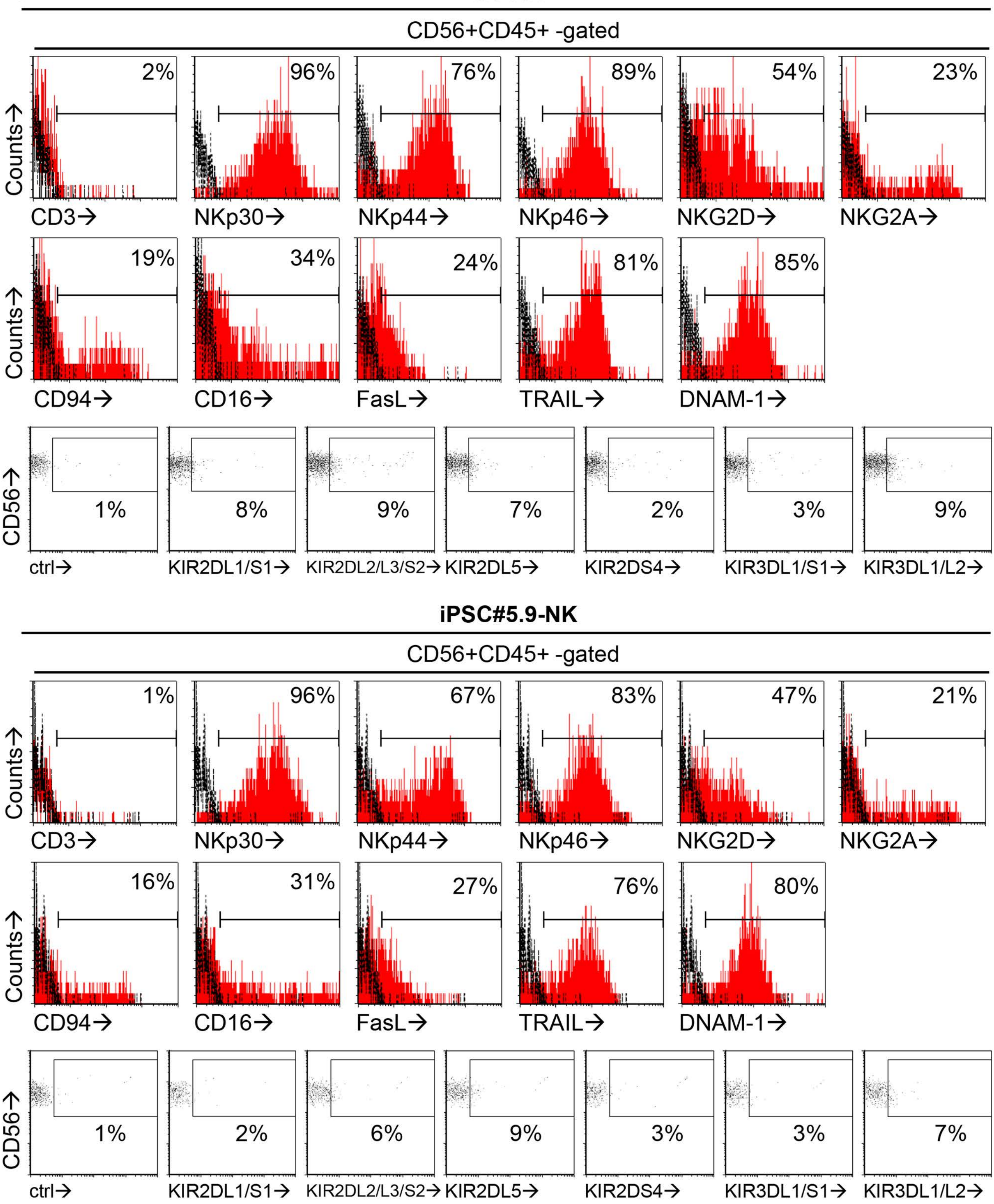

Fig. S2: Phenotype of NK cells generated from hESCs and fibroblast-derived iPSCs. A hESC line, H1 and a fibroblast-derived iPSC line, iPSC#5.9 were used to generate NK cells.

Table S1: A Cytokine Cocktail Improved Purity and Yield of PBC-iPSC-NK Cells

Cytokines	SCF, FLT3L<br>+ IL-7	SCF, FLT3L<br>+ IL-15	SCF, FLT3L<br>+ IL-7, IL-15
**% of<br>CD56+<br>CD45+<br>(purity)**	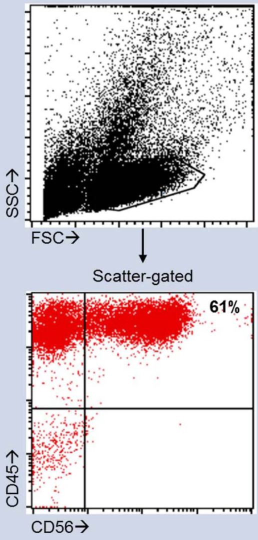	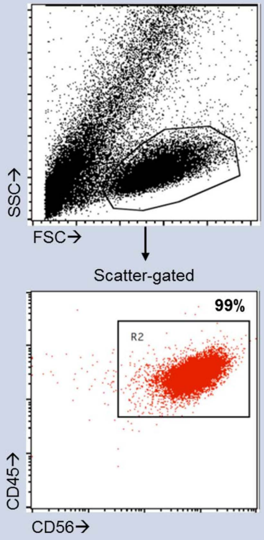	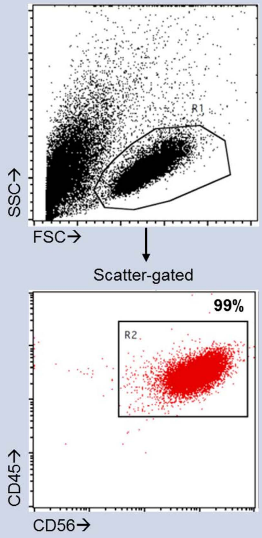
**# of<br>CD56+<br>CD45+<br>(yield)**	$0.21 \times 10^6 \pm 0.18 \times 10^6$<br>per 3×10^6 iPSCs<br>(mean ± SD, n=4, d40)	$0.75 \times 10^6 \pm 0.43 \times 10^6$<br>per 3×10^6 iPSCs<br>(mean ± SD, n=3, d40)	$7.93 \times 10^6 \pm 0.18 \times 10^6$<br>per 3×10^6 iPSCs<br>(mean ± SD, n=6, d40)

Table S2: KIR Genotype and mRNA Expression of PBC-iPSC-NK Cells

KIR gene		2DL1	2DL2	2DL3	2DL4	2DL5all	2DL5A	2DL5B	2DS1	2DS2	2DS3	2DS4del	2DS4ins	2DS5	3DL1	3DL2	3DL3	3DS1	2DP1	3DP1
PB-NK (Donor 4)	KIR genotype	+	+	+	+	-	-	-	-	-	-	+	+	-	+	+	+	-	+	+
	KIR mRNA expression	+	+	+	+	-	-	-	-	-	-	+	+	-	+	+	-	-	+	+
PBC-iPSC#9-NK (Donor A)	KIR genotype	+	+	+	+	-	-	-	-	+	-	+	-	-	+	+	+	-	+	+
	KIR mRNA expression	-	-	-	+	-	-	-	-	-	-	-	-	-	-	-	-	-	-	-
PBC1-iPSC#4-NK (Donor B)	KIR genotype	+	-	+	+	+	+	+	+	-	-	+	-	+	+	+	+	+	+	+
	KIR mRNA expression	-	-	-	+	-	-	-	-	-	-	-	-	-	-	-	-	-	-	-
PBC2-iPSC#12-NK (Donor C)	KIR genotype	-	-	+	+	-	-	-	-	-	-	+	+	-	-	+	+	-	+	+
	KIR mRNA expression	-	-	-	+	-	-	-	-	-	-	+	+	-	-	-	-	-	+	-

Table S3: Comparison of Previous and Current Technologies for Production of NK Cells from hPSCs

Color Code		Pro	Con		Neutral
		Previous Technology Described by Knorr et al, 2013			Current Technology Presented by Zeng et al, 2017
Protocols		**Protocol A - Feeder-dependent protocol #1:** stromal cells M210-B4 for stage I; stromal cells EL08-1D2 for stage II.	**Protocol B - Feeder-dependent protocol #2:** "spin EBs" for stage I; stromal cells EL08-1D2 for stage II; expansion with aAPCs for stage III.	**Protocol C - Feeder-free protocol:** "spin EBs" for stage I; feeder-free method for stage II; expansion with aAPCs for stage III.	**Protocol D - Feeder-dependent protocol:** stromal cells OP9 for stage I; stromal cells OP9-DLL1 for stage II; expansion with aAPCs for stage III.
Starting hPSCs	hESCs	H1, H9	H9	H9	H1
	Fibroblast-derived iPSCs	BJ1-iPSC, DRiPS16	not tested	not tested	iPSC#5.9
	UCB-derived iPSCs	UCBiPS7	UCBiPS7	not tested	not tested
	PBC-derived iPSCs	not tested	not tested	not tested	PBC-iPSC#9, PBC1-iPSC#4, PBC2-iPSC#12 [Comment: Generation of GMP-ready iPSCs from PBCs is "a rountine procedure that is not novel". However, the feasibility to use these PBC-derived GMP-ready iPSCs to generate NK cells has never been demonstrated previously.]
Stage I: Method for differentiating hPSCs to precursor cells	Cell processing	**Feeder-dependent method:** Coculture hPSCs with stromal cells M210-B4. [Comment: "the use of murine stromal layers does not absolutely prohibit clinical translation if master cells banks are used." - a direct quote from Knorr et al, 2013]	**"Spin EB" method:** Step 1 - Adaptation of hPSCs to TrypLE: Passage hPSCs using TrypLE on mEFs (mouse embryonic fibroblasts) for at least 10 passages; Step 2 - Spin EB formation: Seed TrypLE-passaged hPSCs on 96-well plate at 3000 cells/well; spin to aggregate the cells; culture to form EBs. [Comments: 1. mEFs are mouse cells. Thus, the described spin EB method is not a xeno-free process; 2. Spin EB formation is laborious.]		**Feeder-dependent method:** Coculture hPSCs with stromal cells OP9. [Comment: "the use of murine stromal layers does not absolutely prohibit clinical translation if master cells banks are used." - a direct quote from Knorr et al, 2013]
	Medium	RPMI1640 + 15% FBS [Comment: There are gamma-irradiated GMP-grade FBS from different manufacturers such as Lonza, Gibco and Hyclone, which can be used for GMP-complying cell culture.]	Step 1 - Adaptation of hPSCs to TrypLE: Undescribed medium; Step 2 - Spin EB formation: BPEL medium + SCF, BMP-4 and VEGF. [Comments: 1. Undescribed medium was used for adaptation; not sure if it is xeno-free; 2. BPEL (bovine serum albumin polyvinyl alchohol essential lipids) medium contains bovine serum albumin. Thus, the described spin EB method is not a xeno-free process.]		αMEM + 20% FBS [Comment: There are gamma-irradiated GMP-grade FBS from different manufacturers such as Lonza, Gibco and Hyclone, which can be used for GMP-complying cell culture.]
	Duration	21 days	Step 1 - Adaptation of hPSCs to TrypLE: at least 10 passages (~10 weeks); Step 2 - Spin EB formation: 11 days. [Comment: Knorr et al. have used a laborious TrypLE adaptation approach. This approach is not necessary for hPSC aggregation, since the inclusion of ROCK inhibitor in the medium is sufficient for any hPSC to form aggregates in U-bottom 96-well plates following TrypLE dissociation. However, it remains to be tested whether the pretreatment with ROCK inhibitor will affect the differentiation of hPSCs into NK cells.]		12 days [Comment: Protocol D has the shortest duration for stage I comparing with Protocol A, B and C.]
	Precursor cell processing	Sorting CD34+CD45+ cells	No cell sorting required		No cell sorting required
Stage II: Method for differentiating precursor cells to NK cells	Cell processing	**Feeder-dependent method:** Coculture sorted CD34+CD45+ cells with stromal cells EL08-1D2. [Comment: "the use of murine stromal layers does not absolutely prohibit clinical translation if master cells banks are used." - a direct quote from Knorr et al, 2013]	**Feeder-dependent method:** Transfer 6 wells of spin EBs from 96-well plate into one well of 24-well plate pre-seeded with stromal cells EL08-1D2. [Comment: It is laborious for scale-up production.]	**Feeder-free method:** Transfer 6 wells of spin EBs from 96-well plate into one uncoated well of 24-well plate. [Comment: It is laborious for scale-up production.]	**Feeder-dependent method:** Coculture harvested differentiated cells with stromal cells OP9-DLL1. [Comment: "the use of murine stromal layers does not absolutely prohibit clinical translation if master cells banks are used." - a direct quote from Knorr et al, 2013]
	Medium	Unknown medium + NK cell initiaiting cytokines. [Comment: Undescribed medium was used, not sure if it is xeno-free.]			αMEM + 20% FBS + SCF, FLT3L, IL-7, IL-15 [Comment: There are gamma-irradiated GMP-grade FBS from different manufacturers such as Lonza, Gibco and Hyclone, which can be used for GMP-complying cell culture.]
	Duration	28-35 days			28-35 days
hPSC-derived NK cells (before expansion)	Yield	?, not described	~1e6 NK cells per 1.8e4 H9 cells (Fig. 3A). [Comment: Only H9 cells were used to test Protocol B. It is not sure whether Protocol B is applicable to other hPSC lines/sources.]	~0.72e6 NK cells per 1.8e4 H9 cells (Fig. 3A). [Comment: Only H9 cells were used to test Protocol B. It is not sure whether Protocol B is applicable to other hPSC lines/sources.]	15e6 NK cells per 3e6 PBC-iPSCs [Comment: These NK cells can be further expanded by up to 74-fold and the number of expanded NK cells (1.1e9) is enough for clinical application.]
	Purity	?, not described	?, not described [Comment: As estimated from Fig. 2C (the FSC v CD56 dot plots for ES-derived NK cells +EL08 and UCBiPS-derived NK cells +EL08), the purities seemed reasonable.]	?, not described. [Comment: As estimated from Fig. 2C (FSC v CD56 dot plot of ES-derived NK cells -EL08), there was a substantial CD56-negative population. The derived population might not be high-purity.]	up to 99%
	Phenotype	high-level KIR expression (Fig. 1C and Fig. 2C). [Comment: Binding of KIR ligands to KIRs suppresses cytotoxicity of donor NK cells against patient's cancer cells. To improve clinical outcome, elaborately selecting an NK cell donor for a particular patient to obtain a KIR-HLA mismatch in an anti-cancer direction is critical. Besides using NK cells derived from a KIR-HLA mismatched donor, a blocking anti-KIR antibody has also been used in clinical trials to reduce inhibition of NK cells via KIR signaling. These suggest that NK cells with high-level KIR expression may only be applicable in a certain group of patients and high-level KIR expression is not a desirable feature for "off-the-shelf" application.]			KIR-negative. [Comment: A unique KIR-negative phenotype renders PBC-iPSC-NK cells unrestricted by reciepient's HLA genotypes and thus potent cytotoxicity against cancer cells. Since KIR-based therapeutic intervention is unnecessary, PBC-iPSC-NK cells may serve as a universal "off-the-shelf" cell source for many recipients.]
	Cytoxicity against NK-sensitive K562 cells (Potencies of NK cells generated using various protocols and PB-NK are compared.)	NK cells generated from Protocol A only killed 37% of K562 cells at E:T ratio of 10 (supplemental Fig. 2). [Comment: NK cells generated with Protocol A are not potent.]	NK cells generated from Protocol B and Protocol C killed up to 75% of K562 at E:T ratio of 10 (Fig. 2D). [Potency ranking: Protocol B ≈ Protocol C ≈ PB-NK.]		NK cells generated from Protocol D killed up to 90% of K562 at E:T ratio of 10. [Potency ranking: Protocol D > Protocol B ≈ Protocol C ≈ PB-NK > Protocol A]
	Cytotoxicity against multiple myeloma cell lines	not tested	Potency ranking: PB-NK > Protocol B > Protocol C (Fig. 4B). [Comment: NK cells generated from Protocol C have the worse potency among three tested NK cell populations. This is further supported by the IFN-γ and CD107a data (Fig. 4A).]		not tested

	Cytotoxicity against pancreatic cancer cell lines	not tested	Potency ranking: PB-NK > Protocol B (Fig. 4B)	not tested	not tested
Stage III: Method to expand hPSC-derived NK cells	Cell processing	not tested	H9-derived NK cells were cocultured with 9.mbIL-21 aAPC with a aAPC:NK ratio of 2:1 on day 0 and with a ratio of 1:1 weekly thereafter until d70. **[Comments: 1. Laborious cell processing: There were 10 cocultures required during the 70-day process; 2. Requirement of a large and undefined number of aAPCs: Need to prepare aAPC every week for 10 weeks and the number of required aAPC will increase weekly with the expansion of NK cells; 3. High risk of contamination due to long-term cell culture.]**		PBC-iPSC-derived NK cells were cocultured with K562-mbIL15-41BBL with a aAPC:NK ratio of 10:1 on day 0. Expanded NK cells are harvested between day 9 -14. **[Comment: 1. Easy cell processing: Only need one coculture at the beginning; 2. Requirement of a small and defined number of aAPCs: Only need to prepare aAPCs once; 3. Low risk of contamination due to short-term cell culture.]**
	Medium	not tested	RPMI1640 + 15% FBS. **[Comment: There are gamma-irradiated GMP-grade FBS from different manufacturers such as Lonza, Gibco and Hyclone, which can be used for GMP-complying cell culture.]**		CellGro SCGM serum-free medium + 10% FBS. **[Comment: There are gamma-irradiated GMP-grade FBS from different manufacturers such as Lonza, Gibco and Hyclone, which can be used for GMP-complying cell culture.]**
	Duration	not tested	70 days		9 - 14 days
Expanded hPSC-derived NK cells	Expansion fold	not tested	100 to 1000-fold in 70 days		up to 74-fold in 14 days
	Clinical-scale Production (From hPSCs --> NK cells -> expanded NK cells)	not tested	0.25e6 H9 cells -- (x56.8) --> 14e6 NK cells -- (x100) --> 1.4e9 **in 116 days. [Comment: Clinical-scale production is achievable through long-term expansion.]**	0.25e6 H9 cells -- (x40.4) --> 10e6 NK cells -- (x100) --> 1e9 **in 116 days. [Comment: Clinical-scale production is achievable through long-term expansion.]**	3e6 PBC-iPSCs -- (x5) --> 15e6 NK cells -- (x74) --> 1.1e9 **in 61 days. [Comment: Clinical-scale production is achievable through short-term expansion.]**
	Purity	not tested	up to 99%		up to 99%
	Phenotype	not tested	high-level KIR expression (Fig. 5B). **[Comment: Binding of KIR ligands to KIRs suppresses cytotoxicity of donor NK cells against patient's cancer cells. To improve clinical outcome, elaborately selecting an NK cell donor for a particular patient to obtain a KIR-HLA mismatch in an anti-cancer direction is critical. Besides using NK cells derived from a KIR-HLA mismatched donor, a blocking anti-KIR antibody has also been used in clinical trials to reduce inhibition of NK cells via KIR signaling. These suggest that NK cells with high-level KIR expression may only be applicable in a certain group of patients and high-level KIR expression is not a desirable feature for "off-the-shelf" application.]**		KIR-negative. **[Comment: A unique KIR-negative phenotype renders PBC-iPSC-NK cells unrestricted by reciepient's HLA genotypes and thus potent cytotoxicity against cancer cells. Since KIR-based therapeutic intervention is unnecessory, PBC-iPSC-NK cells may serve as a universal "off-the-shelf" cell source for many recipients.]**
	Cytotoxicity against NK-sensitive K562 cells	not tested	15% killing (vs 62% killing before expansion) at E:T ratio of 2.5; 60% killing (vs 75% killing before expansion) at E:T ratio of 10 (Fig. 5D). **[Comment: This decrease in potency suggests that the NK cells are funtionally exhausted after prolonged expansion.]**		up to 65% killing (vs up to 35% killing before expansion) at E:T ratio of 2; up to 90% killing (vs up to 80% killing before expansion) at E:T ratio of 10. **[Comment: This increase in potency suggests that the NK cells are more funtionally mature after short-term expansion.]**
	Cytotoxicity against other cancer cell lines	not tested	not tested		effiently kill a wide range of solid tumor cell lines; more efficient than PB-NK (Fig. 6). **[Comment: The high potency of expanded NK cells is likely due to: (a) the KIR-negative phenotype and (b) the short-term expansion of NK cells.]**
