# Supplementary material for: Generation of “Off-the-Shelf” Natural Killer Cells from Peripheral Blood Cell-Derived Induced Pluripotent Stem Cells
Source: Stem Cell Reports. 2017 Nov 22;9(6):1796–812. doi: 10.1016/j.stemcr.2017.10.020 (PMC5785702; doi:10.1016/j.stemcr.2017.10.020)
Supplement: Document S2. Article plus Supplemental Information [file mmc2.pdf]

Stem Cell Reports
Article
ISSCR
OPEN ACCESS

Generation of "Off-the-Shelf" Natural Killer Cells from Peripheral Blood Cell-Derived Induced Pluripotent Stem Cells

Jieming Zeng,[1,*] Shin Yi Tang,[1,2] Lai Ling Toh,[1] and Shu Wang[1,2,*]
[1]Institute of Bioengineering and Nanotechnology, 31 Biopolis Way, The Nanos, #09-01, Singapore 138669, Singapore
[2]Department of Biological Sciences, National University of Singapore, Singapore 117543, Singapore
*Correspondence: jmzeng@ibn.a-star.edu.sg (J.Z.), swang@ibn.a-star.edu.sg (S.W.)
https://doi.org/10.1016/j.stemcr.2017.10.020

SUMMARY

Current donor cell-dependent strategies can only produce limited "made-to-order" therapeutic natural killer (NK) cells for limited patients. To provide unlimited "off-the-shelf" NK cells that serve many recipients, we designed and demonstrated a holistic manufacturing scheme to mass-produce NK cells from induced pluripotent stem cells (iPSCs). Starting with a highly accessible human cell source, peripheral blood cells (PBCs), we derived a good manufacturing practice-compatible iPSC source, PBC-derived iPSCs (PBC-iPSCs) for this purpose. Through our original protocol that excludes CD34+ cell enrichment and spin embryoid body formation, high-purity functional and expandable NK cells were generated from PBC-iPSCs. Above all, most of these NK cells expressed no killer cell immunoglobulin-like receptors (KIRs), which renders them unrestricted by recipients' human leukocyte antigen genotypes. Hence, we have established a practical "from blood cell to stem cells and back with less (less KIRs)" strategy to generate abundant "universal" NK cells from PBC-iPSCs for a wide range of patients.

INTRODUCTION

Natural killer (NK) cells are innate lymphocytes that kill malignant cells and virus-infected cells (Domogala et al., 2015; Miller, 2013). Target recognition and activation of NK cells depend on an array of activating receptors and inhibitory receptors, which entirely differ from the major histocompatibility complex-restricted T cell receptor (TCR)-dependent mechanism of T cells (Moretta et al., 2014). Thus, it is feasible to use allogeneic NK cells to treat cancers without causing graft-versus-host diseases (GVHDs) (Leung, 2014; Lim et al., 2015), and this effectively expands the cell sources for NK cell therapy beyond an autologous one.

In current clinical trials, large dosages of NK cells ranging from 5×10^6 to 5×10^7/kg body weight have been used (Lapteva et al., 2014). One typical approach to generate such large amounts of allogeneic NK cells is enrichment of NK cells from donor-derived leukapheresis products. Different protocols have been established to make NK cell products through immunemagnetic depletion of T and B cells and selection of CD56+ cells (Koehl et al., 2013). Since residual T and B cells may cause GVHDs and passenger B lymphocyte-mediated complications, respectively, purities of such products are especially crucial for their applications in allogeneic settings. However, to generate high-purity NK cell products, a prolonged manufacturing process is necessary, which not only compromises recovery of NK cells but also their viability and potency. With a low recovery rate, obtaining sufficient NK cells from a single leukapheresis product is difficult, not to mention the limited availability of donor-derived leukapheresis products. Another popular approach to make NK cell products is expansion of NK cells from peripheral blood mononuclear cells (PBMCs) using feeder cells, such as K562 cells modified with membrane-bound molecules such as interleukin-15 (IL-15) and 4-1BB ligand (K562-mbIL15-41BBL) (Fujisaki et al., 2009). These feeder cells can rapidly expand NK cells from PBMCs by 21.6-fold in 7 days (Fujisaki et al., 2009), or from cryopreserved apheresis products by 70-fold in 8 days (Lapteva et al., 2014). Yet, the purities of such short-term cultured NK cells are about 60%–70%, and further expansion up to 21 days or enrichment of NK cells is still required to achieve the purities needed for allogeneic use (Fujisaki et al., 2009; Lapteva et al., 2014). Although the above-mentioned approaches are currently being used to generate NK cell products, the scalabilities of such manufacturing strategies are poor. Like any other donor cell-dependent manufacturing processes, generating NK cell products from primary cells of various donors is difficult to be standardized due to the variable starting materials. It demands specialized facilities and skills, complicated logistics, and high operation cost, which limit the availability of NK cell therapy to a few particular agencies.

Along with the difficulties during manufacturing, selecting a suitable donor of NK cells for a particular patient to improve clinical outcome is another hurdle faced by current strategies for NK cell production (Benson and Caligiuri, 2014; Leung, 2014; Thielens et al., 2012). NK cells express clonally distributed inhibitory receptors known as killer cell immunoglobulin-like receptors (KIRs) (Parham, 2005; Thielens et al., 2012). Each individual KIR recognizes a specific human leukocyte antigen (HLA) class I molecule known as KIR ligand, e.g., KIR2DL1 binds HLA-C2,

This is an open access article under the CC BY license (http://creativecommons.org/licenses/by/4.0/).

KIR2DL2 and KIR2DL3 bind HLA-C1, KIR3DL1 binds HLA-Bw4, and KIR3DL2 binds HLA-A3 and HLA-A11 (Thielens et al., 2012). Binding of KIR ligands to inhibitory KIRs suppresses cytotoxicity of NK cells. To alleviate such inhibition on NK cells and thus to enhance their cytotoxicity against a patient's cancer cells, elaborately selecting an NK cell donor for that particular patient to obtain a KIR-HLA mismatch in an anti-cancer direction is critical (Benson and Caligiuri, 2014; Leung, 2014; Murphy et al., 2012; Thielens et al., 2012). This selection is based on donor KIR typing and recipient HLA typing. A donor is suitable if an inhibitory KIR is present in the donor but the KIR ligand is absent in the recipient (Leung, 2014). The involvement of both KIR and HLA, two highly diverse gene families in human immune system, decides that current donor cell-dependent manufacturing platforms can only produce "custom-made" NK cell products for limited patients instead of "off-the-shelf" ones for a wide range of patients. Therefore, it is imperative to explore an alternative manufacturing strategy that circumvents these aforementioned issues.

In the age of pluripotency, human pluripotent stem cells (hPSCs), especially induced pluripotent stem cells (iPSCs), have emerged as a reliable and standardizable starting material to produce immune cells such as dendritic cells (Zeng et al., 2012, 2015; Zeng and Wang, 2014) and NK cells (Knorr et al., 2013; Woll et al., 2005, 2009). Although a couple of studies have shown the generation of NK cells from hPSCs, these existing protocols require procedures that are unsuitable for large-scale production, e.g., enriching CD34+ cells (Woll et al., 2005, 2009), forming spin embryoid bodies (EBs) (Knorr et al., 2013) and heavily relying on long-term NK cell expansion that lasts more than 2 months (Knorr et al., 2013). Moreover, using these current methods, the resulting NK cells express high-level KIRs (Knorr et al., 2013; Woll et al., 2005, 2009), which restrict their applications to recipients of certain HLA typing. Up to now, a robust good manufacturing practice (GMP)-compatible protocol that consistently generates NK cells from various sources of hPSCs is yet to be reported. To facilitate and contribute to the development of a GMP-ready protocol in the future, we designed and demonstrated a holistic manufacturing scheme to mass-produce NK cells from hPSCs. Starting with a highly accessible human cell source, peripheral blood cells (PBCs), we generated a GMP-compatible hPSC source, PBC-derived iPSCs (PBC-iPSCs) to produce NK cells. Through our original protocol that excludes CD34+ cell enrichment and spin EB formation, high-purity functional and expandable NK cells were generated from PBC-iPSCs. Above all, most of these PBC-iPSC-derived NK (PBC-iPSC-NK) cells expressed no KIRs, which renders them unrestricted by recipients' HLA genotypes. These "HLA-unrestricted" NK cells may serve as a universal "off-the-shelf" NK cell source for many recipients.

RESULTS

Generation of NK Cells from hPSCs

To establish a robust and practical protocol for NK cell production from hPSCs (Figure 1A), we started with a classical hPSC source, the widely used human embryonic stem cell (hESC) line, H1 (Figure 1B). In stage one of this two-stage protocol, to induce hematopoietic differentiation, H1 cells were co-cultured with overgrown OP9 cells, a bone marrow stromal cell line (Figure 1A). On day 12 of co-culture, many differentiated colonies appeared (Figure 1C), and small populations of CD34+ cells (Figure 1J) were consistently observed. In stage two, to induce lymphoid commitment, the differentiated cells were harvested and co-cultured with OP9-DLL1 cells, a modified OP9 cell line expressing Notch ligand Delta-like-1 (DLL1), on a weekly basis in the presence of stem cell factor (SCF), Fms-related tyrosine kinase 3 ligand (FLT3L), and IL-7 (Figure 1A). Seven days after first co-culture with OP9-DLL1 (day 19), most differentiated cells still grew as adherent cells (Figure 1D); after second co-culture (day 26), some bright, round, and semi-attached cells started to pop out (Figure 1E); after third co-culture (day 33) these cells detached and assembled in the center of culture plate well (Figures 1F and 1G); after fourth co-culture (day 40), more suspension cells appeared (Figures 1H and 1I). Morphologically, these suspension cells were small, round, and bright lymphoid cells (Figures 1G and 1I); phenotypically, they appeared as a defined population on scatter plots (Figures 1K and 1L); most were CD45+ CD56+, but CD3− TCRαβ− CD4− CD8− (Figures 1K and 1L), which is typical for NK cells. Thus, by going through hematopoietic differentiation and lymphoid commitment, we established a practical protocol to generate NK cells from hPSCs without employing CD34+ cell enrichment or spin EB formation.

Generation of PBC-iPSCs for Production of NK Cells

To further facilitate NK cell production from hPSCs under GMP, it is helpful to start with a highly accessible hPSC source that is GMP compatible. Although hESCs, fibroblast-derived iPSCs, and umbilical cord blood CD34+ cell-derived iPSCs have been used to generate NK cells (Knorr et al., 2013; Woll et al., 2005, 2009), these hPSC sources are not ideal for GMP. Instead, PBC-iPSCs are a more accessible and practicable option from the standpoint of manufacturing because the starting material PBCs are very amenable to GMP, although the use of this hPSC source to produce NK cells remains unexplored. To study such a possibility, we first generated our own iPSC lines from PBCs (donor A) using integration-free Sendai viral vectors carrying the reprogramming factor genes. The resulting PBC-iPSC lines showed typical morphology of hPSCs (Figure 2A). They were further classified into

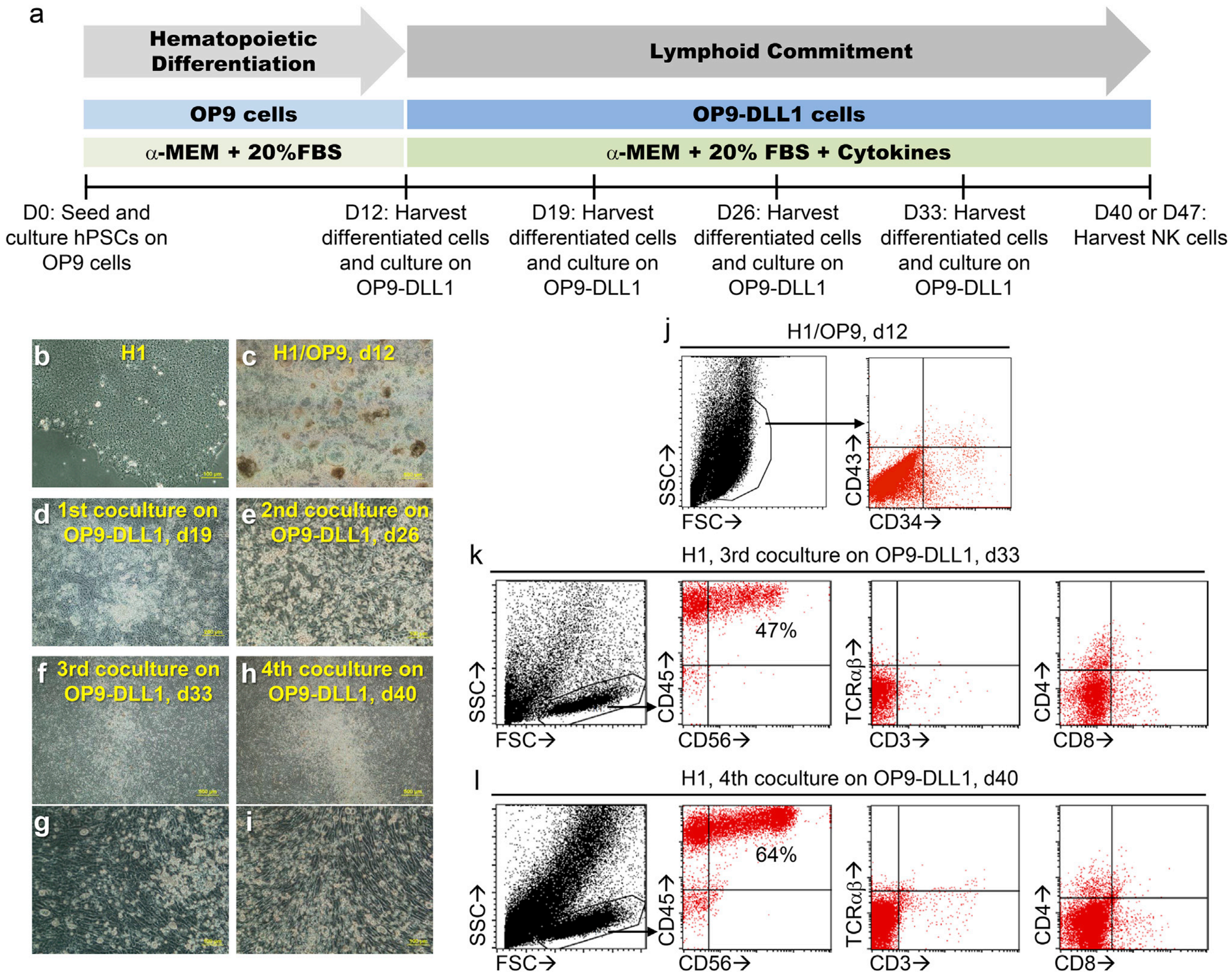

Figure 1. Generation of NK Cells from hPSCs

(A) A schematic of a two-stage protocol for production of NK cells from hPSCs.

(B–I) Morphological changes during differentiation of H1 cells into NK cells. Phase contrast images show (B) undifferentiated H1 cells; (C) H1 and OP9 co-culture, day 12; (D) first differentiated cells and OP9-DLL1 co-culture, day 7 (day 19); (E) second differentiated cells and OP9-DLL1 co-culture, day 7 (day 26); (F and G) third differentiated cells and OP9-DLL1 co-culture, day 7 (day 33); (H and I) fourth differentiated cells and OP9-DLL1 co-culture, day 7 (day 40).

(J–L) Phenotypic changes during differentiation of H1 cells into NK cells. Flow cytometric analysis shows (J) CD34+ cells from H1 and OP9 co-culture, day 12; (K and L) CD56+ CD45+ cells from third (day 33) and fourth (day 40) co-culture on OP9-DLL1.

PBC-iPSC lines derived from T cell or non-T cell by TCRB and TCRG gene clonality assays (Figures 2B and 2C). As shown in Figures 2B and 2C, a PBC-iPSC#8.3 line was identified to be derived from an αβ T cell due to the presence of rearranged TCRβ chain gene, whereas a PBC-iPSC#9 line was from a non-T cell due to the absence of both rearranged TCRβ and TCRγ chain genes.

Next, we examined whether our two-stage protocol for NK cell production is applicable to these PBC-iPSC lines. Using the non-T cell-derived PBC-iPSC#9 line, differentiated cells containing a significant CD34+ population were obtained after a 12-day co-culture with OP9 (Figure 2E). Without sorting CD34+ cells, these differentiated cells were harvested and directly co-cultured with OP9-DLL1. A small CD45+ CD56+ population appeared after the second co-culture (day 26) (Figure 2F); this population became more apparent after the fifth co-culture (day 47), which comprised 61% of the lymphoid cells (Figure 2G). Neither TCRαβ nor CD3 was expressed during the differentiation process; they were undetectable in pluripotent stem cells (Figure 2D), hematopoietic cells (Figure 2E), and lymphoid cells (Figures 2F and 2G). Moreover, there was

no obvious CD4 or CD8 expression in the lymphoid cells (Figures 2F and 2G), confirming that our two-stage protocol could also be used for NK cell production from PBC-iPSCs. Similarly, using the T cell-derived PBC-iPSC#8.3 line, CD34+ cells were generated after a 12-day co-culture with OP9 (Figure 2I); an increasing CD45+ CD56+ population was observed with the progress of lymphoid commitment (Figures 2J and 2K), again proving the robustness of our protocol. This T cell-derived iPSC line itself expressed no TCRαβ (Figure 2H), suggesting the completeness of the reprogramming process. There was no TCRαβ expression in hematopoietic cells either (Figure 2I). However, PBC-iPSC#8.3 did give rise to a distinct CD3+ TCRαβ+ population during lymphoid differentiation (Figures 2J and 2K), although there was no obvious CD4 or CD8 expression (Figures 2J and 2K). The re-expression of rearranged TCRα and TCRβ chain genes in lymphoid cells differentiated from T cell-derived iPSCs is not desirable if the produced cells are to be used in an allogeneic setting. To avoid the potential GVHD caused by the re-expressed TCRαβ, it is crucial to start with PBC-iPSCs derived from non-T cells rather than those from T cells.

To generate more non-T cell-derived PBC-iPSC lines, we used a different protocol to reprogram PBCs (donor B and donor C) into iPSCs, in which PBCs were first cultured in CD34+ cell-enrichment medium for 3 days and episomal reprogramming vectors were then delivered into these pre-treated PBCs via GMP-compatible nucleofection. Using this protocol, we significantly increased the probability of deriving PBC-iPSC lines from non-T cells as verified by TCRB and TCRG gene clonality assays (Figure 2L); nine out of nine verified that PBC1-iPSC lines (donor B) were derived from non-T cells. A possible explanation for this finding is that the proliferating CD34+ cells are more susceptible to reprogramming by nucleofection than the terminally differentiated T cells. In contrast, using the reprogramming protocol with Sendai viral vectors, only five out of nine verified that PBC-iPSC lines (donor A) were from non-T cells. Several confirmed non-T cell-derived PBC-iPSC lines, including PBC-iPSC#9 (donor A), PBC1-iPSC#4 (donor B), and PBC2-iPSC#12 (donor C), were used to produce PBC-iPSC-NK cells in the following studies.

Improving Purity and Yield of NK Cells Produced from Non-T Cell-Derived PBC-iPSCs

In addition to starting with a GMP-compatible hPSC source, improving the purity and yield of NK cells produced from non-T cell-derived PBC-iPSCs will further facilitate the production of this NK cell source under GMP. To this end, we tested whether different cytokine combinations can enhance NK cell commitment. As demonstrated with PBC-iPSC#9 (donor A) (Table S1), using IL-7 together with SCF and FLT3L during lymphoid commitment brought about a purity of 61% and a yield of 0.21×10^6 CD56+ CD45+ cells per 3×10^6 PBC-iPSCs on day 40. The use of IL-15 rather than IL-7 increased the purity to 99% and the yield to 0.75×10^6, while the combined use of IL-7 and IL-15 gave a purity of 99% and a yield of 7.93×10^6 on day 40, which further increased to 15×10^6 on day 47.

Using this optimized cytokine combination, high-purity PBC-iPSC-NK cells were produced from PBC-iPSC#9 (donor A) (Figures 3A–3D). These PBC-iPSC#9-NK cells were so cytotoxic that they killed almost all OP9-DLL after the fifth co-culture (Figure 3A). After harvesting, any potential carried-over OP9 or OP9-DLL1 or cell debris can be further removed by density gradient centrifugation using Ficoll-Paque and overnight culture (Figure 3B). There was no detectable contamination of OP9 or OP9-DLL1, since the whole resulting cell population was positively stained by an anti-human CD45 monoclonal antibody (Figure 3C), which could not stain both OP9 and OP9-DLL1 (Figure S1). These cells expressed no CD3, CD19, or CD14, suggesting that they are not T cells, B cells, or monocytes (Figure 3C). Moreover, they showed typical morphology of NK cells (Figure 3B). They were a homogeneous population as demonstrated by the cell images (Figure 3B) and the scatter plot (Figures 3C and 3D). Most were CD56+ CD45+ CD3−, a typical NK cell phenotype (Figure 3D). More importantly, these cells expressed numerous receptors and surface molecules that are crucial for effector functions of NK cells, including natural cytotoxicity receptors (NKp30, NKp44, and NKp46), activating receptors (NKG2D and DNAM-1), and death-inducing ligands (FasL, TRAIL) (Figure 3D). They also expressed CD16 (Figure 3D), which mediates antibody-dependent cell-mediated cytotoxicity (ADCC) of NK cells. Thus, these PBC-iPSC-NK cells express all the best-characterized activating receptors and surface molecules of NK cells that are implicated in fighting cancer. Besides activating receptors, they expressed inhibitory receptor CD94:NKG2A (Figure 3D), which may prevent over-activation of NK cells. Likewise, using PBC1-iPSC#4 (donor B), we were able to generate PBC1-iPSC#4-NK cells with a similar phenotype (Figure 3E). Hence, a large number of high-purity NK cells can be consistently produced from various PBC-iPSC lines using our optimized two-stage protocol.

Functions of PBC-iPSC-NK Cells

Secreting cytokines such as interferon-γ (IFN-γ) upon stimulation is an important functional feature of NK cells, which can be detected by an enzyme-linked immunospot (ELISPOT) assay. To investigate IFN-γ secretion, PBC-iPSC#9-NK cells were co-cultured with stimulating cancer cells. ELISPOT results (Figures 4A and 4B) showed that K562, an NK cell-sensitive leukemia cell line, was efficient in stimulating IFN-γ secretion by PBC-iPSC#9-NK cells,

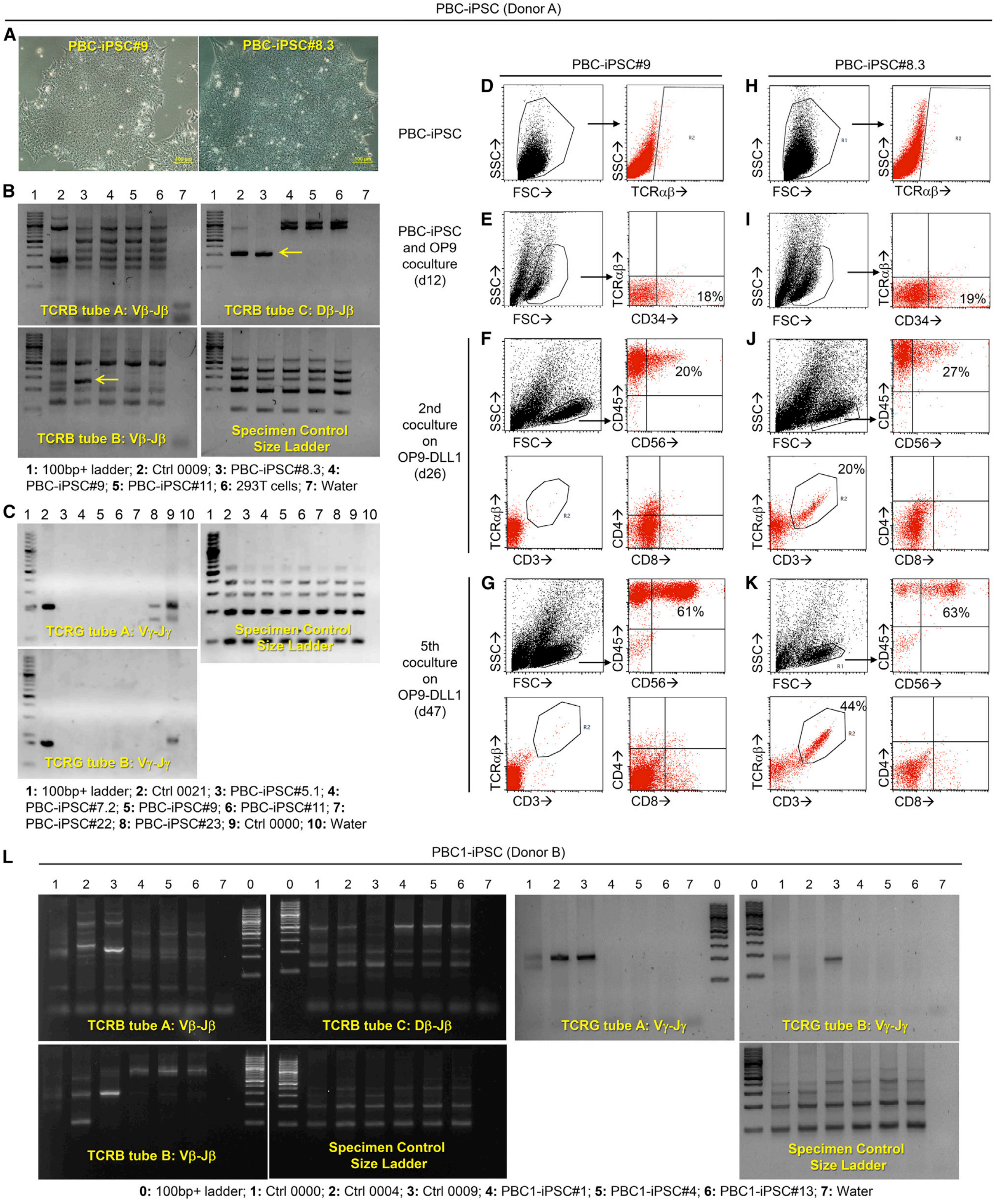

Figure 2. Generation of PBC-iPSCs for Production of NK Cells

PBCs from different donors were used to generate various PBC-iPSC lines. Results were obtained using PBC-iPSC (donor A) lines (A–K) and PBC1-iPSC (donor B) lines (L).

(legend continued on next page)

whereas Raji, an NK cell-resistant lymphoma cell line, was less efficient, suggesting that PBC-iPSC-NK cells are capable of secreting cytokine in response to stimulation.

Cytotoxicity is another hallmark of NK cells, which depends on secretion of cytotoxic molecules such as granzyme B (GrB). ELISPOT results (Figures 4C and 4D) showed that PBC-iPSC#9-NK cells secreted GrB upon stimulation by K562 (Figures 4C and 4D), which further confirms the functional competency of PBC-iPSC-NK cells. Moreover, as demonstrated in Figures 4E and 4F, PBC-iPSC#9-NK cells had a direct killing profile similar to that of primary NK cells. They were able to kill the NK cell-sensitive K562 cells directly, but not the NK cell-resistant Raji cells (Figures 4E and 4F). Interestingly, by exploiting their ADCC function, PBC-iPSC#9-NK cells were still capable of killing Raji cells (Figures 4G and 4H). As shown in Figures 4G and 4H, PBC-iPSC#9-NK cells alone were unable to kill Raji cells; neither with addition of human IgG1 or anti-CD20-mIgG1. With addition of anti-CD20-hIgG1 of various concentrations, however, distinct cytotoxicity against Raji cells appeared. Therefore, PBC-iPSC-NK cells are fully functional in terms of cytokine secretion, direct cytotoxicity, and ADCC.

Expansion of Fresh and Cryopreserved PBC-iPSC-NK Cells

Generating sufficient PBC-iPSC-NK cells is a prerequisite for their translation into clinical use. One possible way is to scale up the differentiation cultures, which, however, is not cost-effective. A more practical way is to expand PBC-iPSC-NK cells using feeder cells. To investigate this possibility, various numbers of PBC-iPSC#9-NK cells were co-cultured with irradiated K562-mbIL15-41BBL cells at an NK cell:feeder cell ratio of 1:10 in gas-permeable G-Rex10 flasks. Results showed that PBC-iPSC-NK cells were expandable (Figures 5A and 5B). Starting with 10^6 PBC-iPSC#9-NK cells, up to 74-fold expansion was achieved on day 9, although no further expansion was observed after extending co-culture to 14 days (Figure 5A). The expanded PBC-iPSC#9-NK cells kept their phenotype apart from the downregulation of CD16 (Figure 5C); functionally, they became more potent, as shown by higher cytotoxicity against K562 cells (Figure 5D). These findings indicate that clinical-scale production of PBC-iPSC-NK cells through expansion with feeder cells is feasible.

Shipping therapeutic cell products from a centralized manufacturing site to a clinical site for injection without compromising the product quality is crucial for the clinical success of live cell products. It is well known that transporting conventional NK cell products in cryopreserved form can significantly reduce their viability and potency (Lapteva et al., 2014), which might also be a problem for PBC-iPSC-NK cells. Indeed, current freeze/thaw procedure significantly affected viability of expanded PBC-iPSC#9-NK cells (Figure 5E), although the cytotoxicity of these cells was partially preserved (Figure 5G, expanded NK→frozen-thawed). To overcome this issue, we proposed a possible solution: at a centralized manufacturing site, PBC-iPSC-NK cells are first produced and cryopreserved (before expansion); the cryopreserved PBC-iPSC-NK cells are then shipped to a clinical site; at the clinical site, the cryopreserved PBC-iPSC-NK cells are then thawed, expanded, and injected. The feasibility of such a solution depends on the expansion capability of cryopreserved PBC-iPSC-NK cells. As shown in Figure 5F (frozen-thawed NK), the cryopreserved PBC-iPSC-NK cells remained expandable. Starting with 10^6 cryopreserved PBC-iPSC-NK cells, up to 38.5-fold expansion was achieved on day 9. Above all, these expanded cryopreserved NK cells had comparable cytotoxicity as those expanded from fresh NK cells (Figure 5G, frozen-thawed NK→expansion versus fresh NK→expansion). These results support that transporting cryopreserved pre-expansion PBC-iPSC-NK cells, followed by their expansion and injection at the clinical site, may provide a practical solution to the logistics of such cell products.

Cytotoxicity of PBC-iPSC-NK Cells against Cancer Cells

To evaluate the cytotoxicity of PBC-iPSC-NK cells against cancer cells, three PBC-iPSC lines, PBC-iPSC#9 (donor A), PBC1-iPSC#4 (donor B), and PBC2-iPSC#12 (donor C), were used to generate PBC-iPSC-NK cells named PBC-iPSC#9-NK cells, PBC1-iPSC#4-NK cells, and PBC2-iPSC#12-NK cells, respectively. After expansion, these PBC-iPSC-NK cells were used for cytotoxicity assay against a wide variety of cancer cell lines: K562 (chronic myelogenous leukemia), SK-OV-3 (ovary adenocarcinoma), SW480 (colorectal adenocarcinoma), HCT-8 (ileocecal colorectal

(A) Morphology of two PBC-iPSC (donor A) lines, PBC-iPSC#9 and PBC-iPSC#8.3.
(B and C) TCRB gene (B) and TCRG gene (C) clonality assays to detect rearranged TCRβ and TCRγ chain genes in PBC-iPSC (donor A) lines. Positive amplified product is indicated by the yellow arrow.
(D–K) Production of NK cells from two PBC-iPSC (donor A) lines, PBC-iPSC#9 and PBC-iPSC#8.3. (D–G) Phenotypic changes during differentiation of non-T cell-derived PBC-iPSC#9 line into NK cells. Flow cytometric analysis shows no TCRαβ expression during differentiation. (H–K) Phenotypic changes during differentiation of T cell-derived PBC-iPSC#8.3 line into NK cells. Flow cytometric analysis shows re-expression of TCRαβ during differentiation.
(L) TCRB gene and TCRG gene clonality assays to detect rearranged TCRβ and TCRγ chain genes in PBC1-iPSC (donor B) lines.

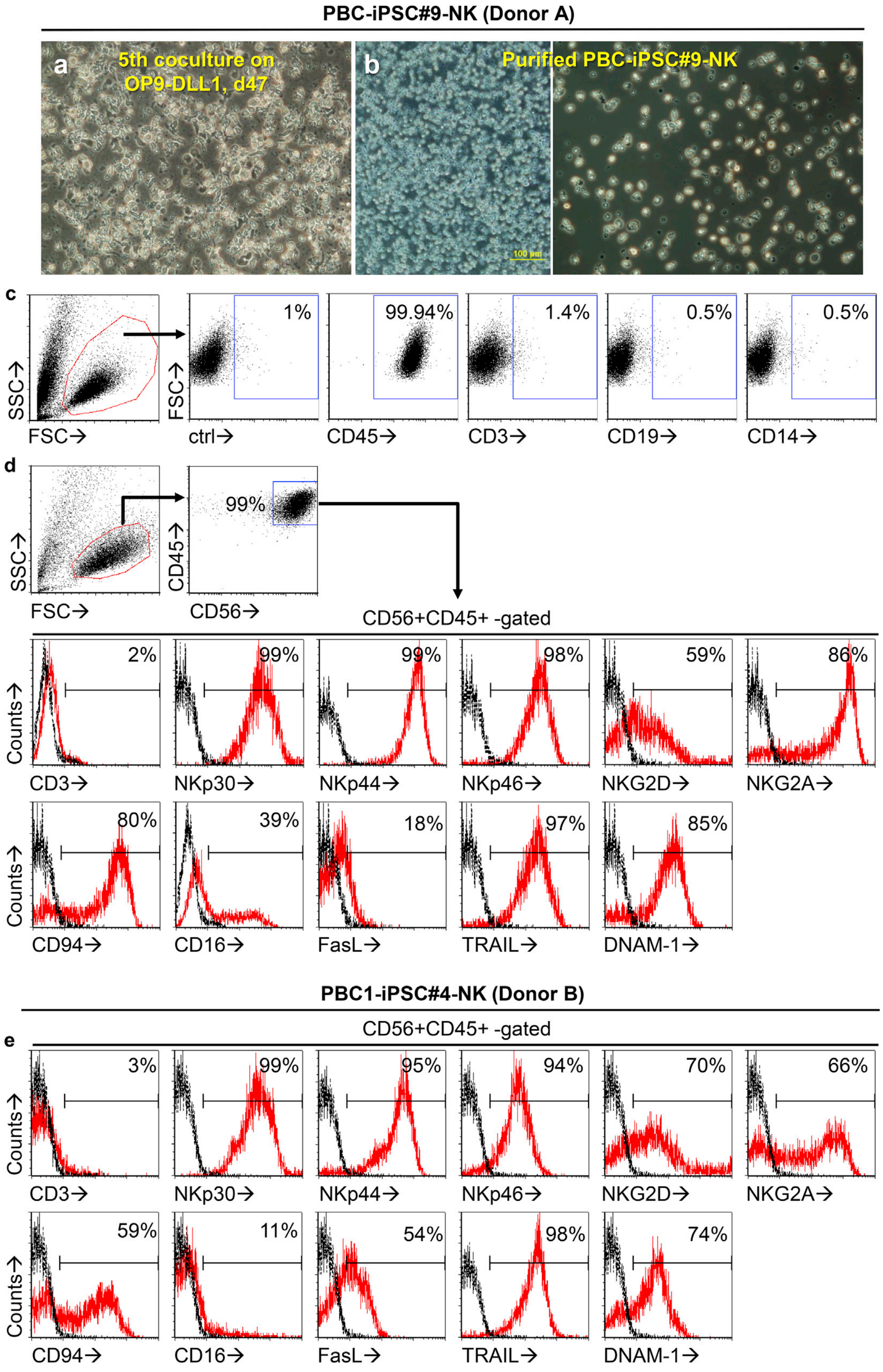

(legend on next page)

adenocarcinoma), MCF7 (breast adenocarcinoma), and SCC-25 (tongue squamous cell carcinoma). Peripheral blood NK (PB-NK) cells expanded from three different donors (donor 1, donor 2, and donor 3) were used as controls. Results showed that all tested PBC-iPSC-NK cells efficiently killed all tested cancer cell lines; cytotoxicity was observed even at very low effector to target (E:T) ratios (Figure 6). When testing against NK cell-sensitive K562 cells, cytotoxicity of PBC-iPSC-NK cells was similar to that of PB-NK cells (Figure 6A); however, against other solid tumor lines, PBC-iPSC-NK cells were consistently more efficient in killing the target cells than PB-NK cells (Figures 6B–6F).

In addition to using existing cancer cell lines, we also derived primary tumor cells from a type of solid tumor, colorectal cancer, for cytotoxicity assay. Characterization of a short-term cultured primary tumor cell line CRC7.4 is shown in Figures 6G–6K. This CRC7.4 line expressed cytokeratin (Figure 6G), but not vimentin (Figure 6H), suggesting the epithelial origin of the cancer cells. Moreover, CRC7.4 had high-level expression of CD133, a cancer stem cell (CSC) marker (Figures 6I and 6K), as well as other CSC markers such as CD44 and EpCAM (Figure 6K). There was little expression of CK20 (Figure 6J), which is usually expressed in goblet cells and enterocytes of the gastrointestinal tract. Such a phenotype indicates that the CRC7.4 line contains a high percentage of CSCs, but little differentiated cancer cells. Cytotoxicity assay showed that PBC-iPSC-NK cells were able to kill these CSC-like cancer cells at the tested E:T ratios, while the expanded PB-NK cells were inefficient at such low ratios (Figure 6L).

KIR Typing of PBC-iPSC-NK Cells

Signaling through inhibitory KIRs inhibits NK cell cytotoxicity. To understand the high anti-tumor efficacy of PBC-iPSC-NK cells, we compared KIR expression of PBC-iPSC-NK cells and PB-NK cells. KIR genotyping showed that donors of these different NK cells had diverse KIR gene contents (Figures 7A, 7C, 7G, and 7I; Table S2). In PB-NK cells, most KIR genes were actively expressed, as verified at the mRNA level (Figure 7B; Table S2) and the protein level (Figure 7K). In PBC-iPSC-NK cells, however, transcription of KIR genes was undetectable, except for KIR2DL4 gene, a framework KIR gene (Figures 7E, 7H, and 7J; Table S2); phenotyping further confirmed this finding, since expression of most KIRs was undetectable on the cell surface by immunostaining with various anti-KIR antibodies, although small populations of KIR2DL2/L3/S2+ cells were observed (Figures 7L, 7M, and 7N). Moreover, the NK cell expansion process did not change KIR expression in PBC-iPSC-NK cells, as demonstrated at the mRNA (Figure 7F) and protein levels (Figures 7O and 7P). These data showed that most PBC-iPSC-NK cells were negative for the widely studied inhibitory KIRs: KIR2DL1, KIR2DL2, KIR2DL3, KIR3DL1, and KIR3DL2. Such an inhibitory KIR-negative phenotype makes PBC-iPSC-NK cells insensitive to inhibition by KIR ligands, which may account for their high anti-tumor cytotoxicity.

To understand whether the hPSC source or our differentiation protocol is responsible for the KIR-negative phenotype, we derived NK cells from two different hPSC sources: an hESC line, H1, and a fibroblast-derived iPSC line, iPSC#5.9, which was generated previously in our lab (Yang et al., 2012) (Figure S2). KIR phenotyping showed that most NK cells derived from these two hPSC sources were also KIR negative (Figure S2). These results suggest that the KIR-negative phenotype is not limited to the NK cells derived from PBC-iPSCs. Other hPSC sources can also be used to generate KIR-negative NK cells using our differentiation protocol.

DISCUSSION

Allogeneic NK cells are currently being used in cancer immunotherapy (Leung, 2014; Lim et al., 2015). To produce a large amount of these cells, donor-derived leukapheresis products or PBMCs are processed through either NK cell enrichment or NK cell expansion (Fujisaki et al., 2009; Koehl et al., 2013; Lapteva et al., 2014). The use of highly variable starting materials makes it difficult to consistently produce NK cell products using such manufacturing strategies. Moreover, these strategies have poor scalability due to limited availability of donor cells. The reliance on donor-derived NK cells also determines that the final products express high-level KIRs. A suitable NK cell donor has to be selected for a particular patient to achieve a KIR-HLA mismatch and thus a better clinical outcome (Benson and Caligiuri, 2014; Leung, 2014; Murphy et al., 2012; Thielens et al., 2012). This

Figure 3. Morphology, Purity, and Phenotype of PBC-iPSC-NK Cells
(A) Morphology of fifth differentiated cells and OP9-DLL1 co-culture, day 7 (day 47) starting with PBC-iPSC#9 (donor A).
(B) Morphology of PBC-iPSC#9-NK cells (donor A) after harvesting and purification by density gradient centrifugation using Ficoll-Paque followed by overnight culture.
(C) Purity of PBC-iPSC#9-NK cells (donor A) as evaluated by flow cytometry.
(D) Phenotype of PBC-iPSC#9-NK cells (donor A).
(E) Phenotype of PBC1-iPSC#4-NK cells (donor B).

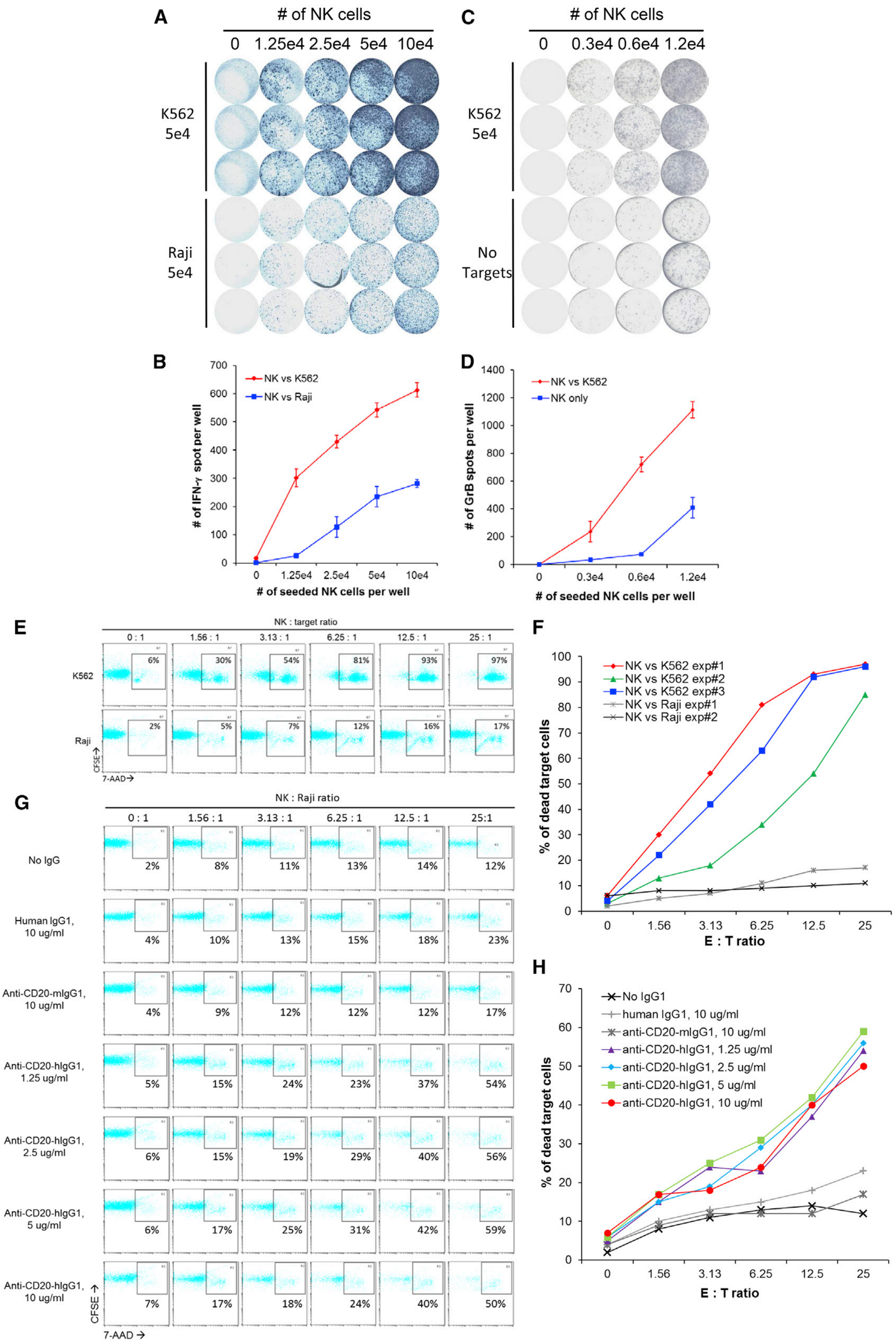

(legend on next page)

requirement of a matched donor, in this case a KIR-HLA mismatched donor, decides that such manufacturing strategies can only produce "made-to-order" NK cell products, which cannot fulfill the urgent needs of cancer patients. All these issues call for a different manufacturing approach that produces "off-the-shelf" NK cell products. To this end, we have demonstrated an overall production scheme: from blood cells to stem cells and back with less (less KIRs), to make such products. This PBC-iPSC-based manufacturing strategy is standardizable and scalable, which produces "off-the-shelf" KIR-negative NK cells that may serve a wide range of recipients disregarding their HLA genotypes in a timely manner.

To implement this production scheme under GMP, it is important to embrace GMP early on by starting with a GMP-compatible hPSC source. Among current hPSC sources, hESCs are a safe option, but their derivations are ethically controversial and their applications are strictly allogeneic. In contrast, iPSCs have no ethical issue and they can be applied in both autologous and allogeneic settings. Various starting cells have been used to derive iPSC lines (Gonzalez et al., 2011). The choice of starting cells affects not only the efficiency and kinetics of reprogramming, but also practicality in deriving GMP-grade iPSCs. Although skin fibroblasts are most commonly used to derive iPSC lines, they are not ideal for GMP. Deriving skin fibroblasts requires collection of skin samples through an invasive punch biopsy and a time-consuming process to grow fibroblasts from skin sample (it takes up to 3 weeks), and doing all these under GMP is a daunting task. Unlike deriving skin fibroblasts, obtaining PBCs is much simpler: it only requires blood sample collection through a routine blood withdrawal and a simple isolation of mononuclear cells using Ficoll-Paque, both of which are GMP ready. Thus, we used PBCs to derive PBC-iPSC lines for NK cell production. It is noteworthy that not all PBC-iPSC lines are created equal in terms of NK cell production. Our findings suggest that while using a T cell-derived PBC-iPSC line, the rearranged TCR genes will re-express, which is not desirable due to the risk of GVHD in allogeneic application. To avoid such a consequence, one should only use non-T cell-derived PBC-iPSC lines. For this reason, we also established a reprogramming protocol using GMP-compatible nucleofection to derive PBC-iPSC lines of non-T cell-origin. With these verified non-T cell-derived lines, we successfully demonstrated the production of NK cells from PBC-iPSCs.

Inducing hematopoietic differentiation of hPSCs is a crucial first step for generating NK cells from hPSCs. One approach is to co-culture hPSCs with bone marrow stromal cells (e.g., S17 and M2-10B4) to obtain CD34+ hematopoietic precursors (Knorr et al., 2013; Woll et al., 2005, 2009). However, it requires co-culture for as long as 21 days and subsequent sorting of CD34+ cells before differentiation into NK cells. This cell-sorting step not only complicates the protocol, but also reduces yield of NK cells because it excludes many other hematopoietic progenitors that are also competent for differentiation into NK cells. Another approach is to form EBs (Tabatabaei-Zavareh et al., 2007) and recently spin EBs (Knorr et al., 2013). Spin EB formation requires a prior time-consuming adaptation of hPSCs to TrypLE digestion for at least ten passages on mouse embryonic fibroblasts, which may not be easy for many hPSC lines. To form spin EBs, TrypLE-adapted hPSCs are seeded onto 96-well plates at a density of 3,000 cells per well and spun down. This is followed by harvesting and seeding spin EBs at a density of six spin EBs per well on 24-well plates for further NK cell differentiation. Technically, spin EB approach is labor-intensive, skill-demanding, and difficult for large-scale production. Inherently, the hematopoietic progenitors generated via spin EB formation are variable and inconsistent, which may result in high variation in later NK cell development. It has been demonstrated that the adaptation of hPSCs to TrypLE digestion is not necessary for hPSC aggregation, since the inclusion of ROCK inhibitor in the medium is sufficient for any hPSCs to form aggregates in U-bottomed 96-well plates following TrypLE dissociation (Lancaster et al., 2013; Nakano et al., 2012). However, it remains to be tested whether the use of ROCK inhibitor will affect the differentiation of hPSCs into NK cells. To develop a practical protocol, we revisited the co-culture approach using OP9, a classical M-CSF-deficient stromal cell line, which has been reliably used to generate dendritic cells from hPSCs in our previous studies (Zeng et al., 2012, 2015; Zeng and Wang, 2014).

Figure 4. Functions of PBC-iPSC-NK Cells

(A and B) IFN-γ secretion by PBC-iPSC#9-NK cells (donor A) upon stimulation with K562 and Raji cells as detected by ELISPOT assay. ELISPOT images (A) and spot counting (mean ± SD, n = 3) (B) are shown.

(C and D) GrB secretion by PBC-iPSC#9-NK cells upon stimulation with K562 cells as detected by ELISPOT assay. ELISPOT images (C) and spot counting (mean ± SD, n = 3) (D) are shown.

(E and F) Cytotoxicity of PBC-iPSC#9-NK cells against K562 and Raji cells as measured by flow cytometry. A representative flow cytometric analysis (E) and a result summary (F) are shown.

(G and H) ADCC of PBC-iPSC#9-NK cells against Raji cells in the presence of anti-CD20 humanized antibody as measured by flow cytometry. A representative flow cytometric analysis (G) and a result summary (H) are shown. These data are representative of three independent experiments.

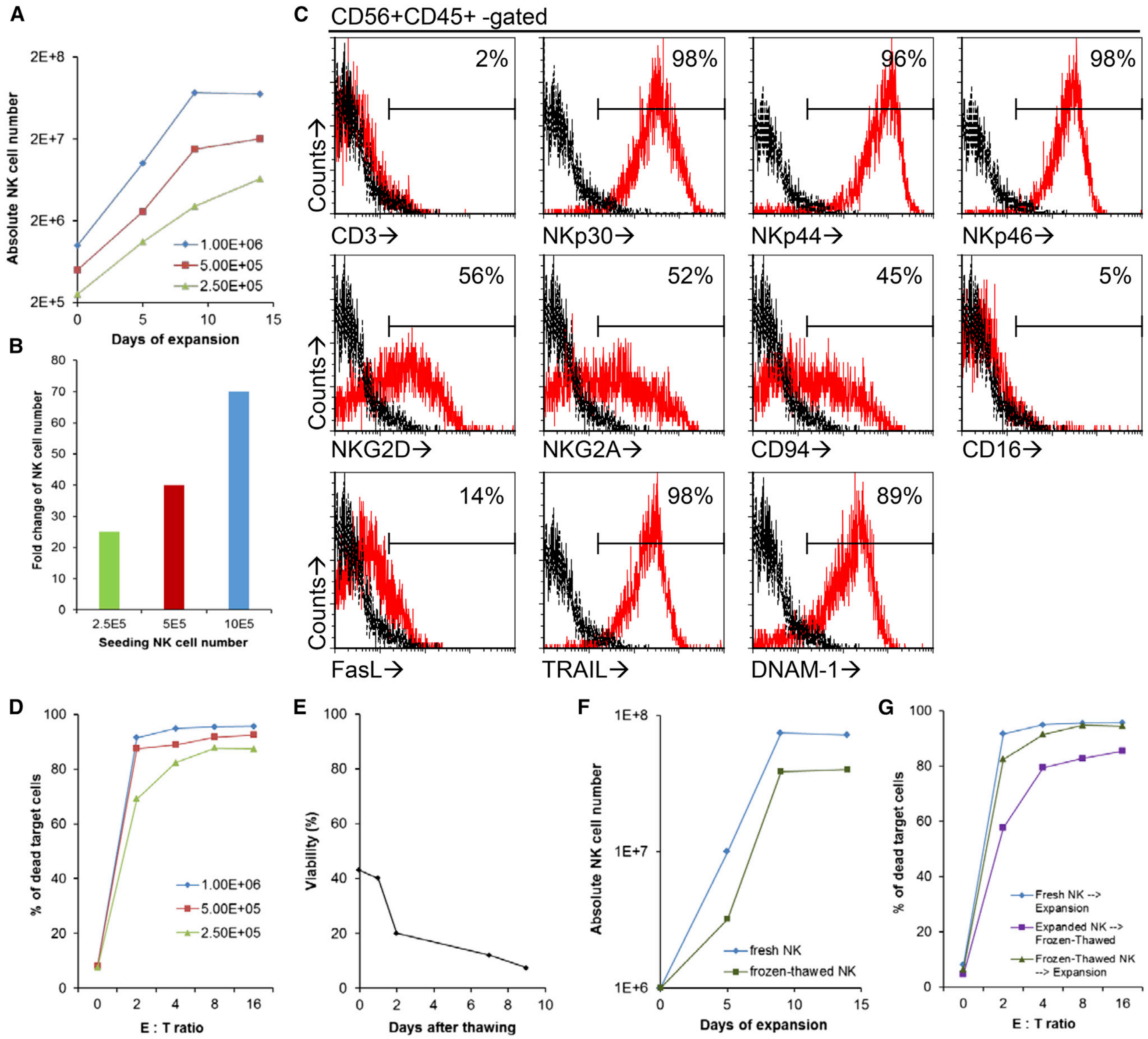

Figure 5. Expansion of Fresh and Cryopreserved PBC-iPSC-NK Cells

(A and B) Expansion of fresh PBC-iPSC#9-NK cells by K562-mbIL15-41BBL in G-Rex10 starting with different NK cell numbers. Absolute numbers of NK cells during a 14-day expansion (A) and fold changes after expansion (B) are shown.

(C and D) Phenotype (C) and cytotoxicity against K562 (D) of fresh PBC-iPSC#9-NK cells after expansion as measured by flow cytometry.

(E) Viability of expanded PBC-iPSC#9-NK cells after freeze/thaw procedure.

(F) Expansion of cryopreserved PBC-iPSC#9-NK cells.

(G) Cytotoxicity of cryopreserved PBC-iPSC#9-NK cells against K562 after expansion. These data are representative of three independent experiments.

By co-culturing with OP9, efficient hematopoietic differentiations of hESCs and PBC-iPSCs have been consistently achieved within 12 days, which is significantly shorter than using other stromal cell lines. Without sorting CD34+ cells, harvested hematopoietic cells can be directly used for further differentiation. Thus, using OP9 to induce hematopoietic differentiation can significantly simplify the manufacturing process.

Promoting lymphoid commitment of hematopoietic progenitors by further co-culturing them with stromal cell lines is the second step for generating NK cells from hPSCs. Although stromal cell lines, such as MS-5 and

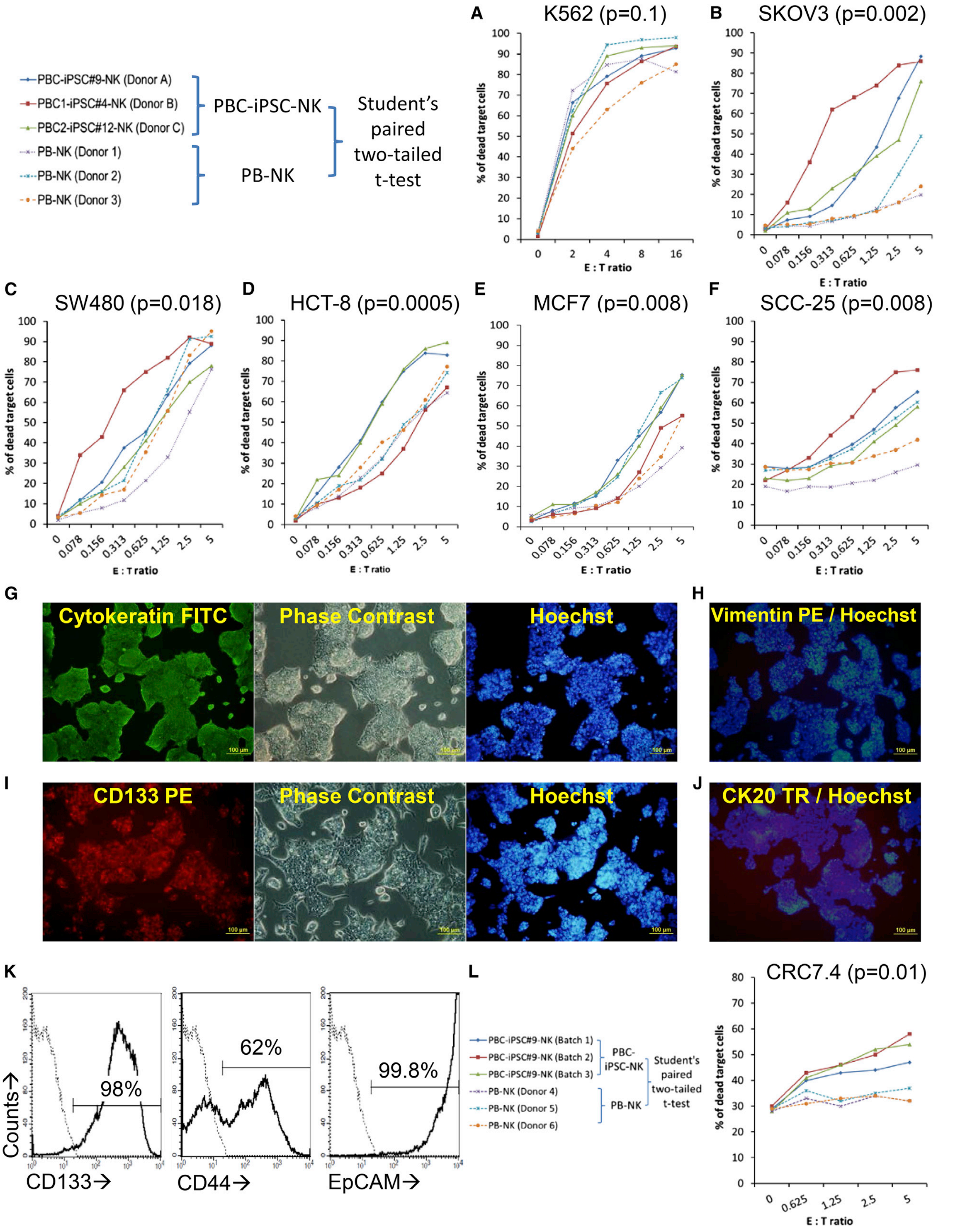

(legend on next page)

AFT024, have been used, the resulting cells were heterogeneous populations containing both CD56+ and CD56− cells (Vodyanik et al., 2005; Woll et al., 2005, 2009). Improved NK development was observed using a stromal cell line EL08-1D2 (McCullar et al., 2008); however, its efficacy was only demonstrated with sorted CD34+ cells or spin EBs (Knorr et al., 2013; Woll et al., 2009). Knorr et al. (2013) also demonstrated that hPSC-derived stromal cells support NK cell development from hematopoietic progenitor cells. However, in terms of robustness, hPSC-derived stromal cells were even less efficient than the EL08-1D2 cells; the formers could only produce NK cells with 76.4% purity from UCB CD34+ cells, while the latter produced 96.7% (Knorr et al., 2013). To provide a robust microenvironment for NK cell development, we used OP9-DLL1, a modified OP9 cell line expressing Notch ligand DLL1, together with an NK cell-promoting cytokine cocktail (SCF, FLT3L, IL-7, and IL-15). This is because activation of Notch signaling pathway is vital for development of innate lymphoid cells (De Obaldia and Bhandoola, 2015). Notch ligand DLL1 suppresses B cell development and promotes NK cell development from human cord blood CD34+ cells (Jaleco et al., 2001). Moreover, DLL1 is able to augment the proliferation of primitive hematopoietic progenitor *in vitro* (Karanu et al., 2001), thus providing a potential benefit to increase the yield of NK cells from hPSCs. In this study, we have demonstrated that, using OP9-DLL1 as feeders, heterogeneous hematopoietic cells harvested from PBC-iPSC/OP9 co-cultures can be directly seeded to generate NK cells. The end products were homogeneous CD56+ CD45+ lymphoid populations.

Hence, through sequential hematopoietic differentiation on OP9 cells and lymphoid commitment on OP9-DLL1 cells, we have established a robust protocol to generate high-purity, functional, and expandable PBC-iPSC-NK cells. Most of these PBC-iPSC-NK cells have a KIR-negative phenotype, which has an interesting implication for their clinical use. Unlike PBC-iPSC-NK cells, conventional donor-derived NK cells express high-level KIRs and require KIR-based therapeutic intervention to improve clinical outcome (Benson and Caligiuri, 2014; Leung, 2014; Murphy et al., 2012; Thielens et al., 2012). Besides using NK cells derived from a KIR-HLA mismatched donor, a blocking anti-KIR antibody that binds KIR2DL1/L2/L3 has also been used in clinical trials to reduce inhibition imposed by HLA-C alleles on NK cells (Benson et al., 2012, 2015; Vey et al., 2012). As a further expansion of this concept, development of a KIR-negative NK cell source may obliterate the need of KIR-based intervention. Without KIR expression, such NK cells are unrestricted by HLA phenotypes of recipients and thus can be developed into universal “off-the-shelf” NK cell products. Practically, it would be difficult to generate such cells by downregulating KIR expression of the KIR-positive donor-derived NK cells due to the complexity of the KIR gene family. However, in this study, we have proved that it is possible to produce such KIR-negative NK cells through *de novo* generation from hPSCs. This is likely resulting from our differentiation protocol rather than the source of hPSCs, since the KIR-negative phenotype was observed in NK cells generated from PBC-iPSCs, as well as hESCs and fibroblast-derived iPSCs. Phenotypically, these hPSC-derived NK cells express most typical receptors and surface molecules of NK cells except KIRs; functionally, they are fully competent: they secrete cytokines, release GrB upon stimulation, and are capable of killing target cells via direct recognition and ADCC. After short-term expansion by feeder cells, these NK cells become more potent in cytotoxicity, but remain KIR negative. This population is similar to the KIR-negative “pseudomature lytic NK cells” derived from human CD34+ cells after prolonged culture with IL-15 (Colucci et al., 2003), and may represent a particular stage of NK cell development. Compared with previous studies that generated KIR-positive NK cells from hPSCs (Knorr et al., 2013; Woll et al., 2005, 2009), one distinct difference is the use of OP9-DLL1 in this study to direct differentiation of precursor cells into NK cells. However, whether activating Notch signaling pathway by DLL1 is responsible for generating the KIR-negative phenotype in the derived NK cells remains to be elucidated.

Our overall approach may facilitate manufacturing of NK cells from hPSCs due to the following technical specifications: (1) our starting material PBC-iPSCs are a highly accessible and GMP-compatible hPSC source;

Figure 6. Cytotoxicity of PBC-iPSC-NK Cells against Cancer Cells
Three different sources of PBC-iPSC-NK cells, including PBC-iPSC#9-NK cells (donor A), PBC1-iPSC#4-NK cells (donor B), and PBC2-iPSC#12-NK cells (donor C), were used for cytotoxicity assay against a wide variety of cancer cell lines: K562 (A), SK-OV-3 (B), SW480 (C), HCT-8 (D), MCF7 (E), and SCC-25 (F). PB-NK cells expanded from three different donors (donor 1, donor 2, and donor 3) were used as controls. (G–I) A short-term cultured primary tumor cell line CRC7.4 was derived from a colorectal cancer sample and characterized by immunostaining (G–J) and flow cytometry (K). These primary tumor cells were then use as target cells to evaluate the cytotoxicity of PBC-iPSC#9-NK cells (L). PB-NK cells expanded from three donors (donor 4, donor 5, and donor 6) were used as controls. Student’s paired two-tailed t test was used to analyze the difference between the specific lysis of target cells by PBC-iPSC-NK cells and that by PB-NK cells. The p values were calculated for each type of target cell and a p value less than 0.05 was considered to be statistically significant. These data are representative of three independent experiments.

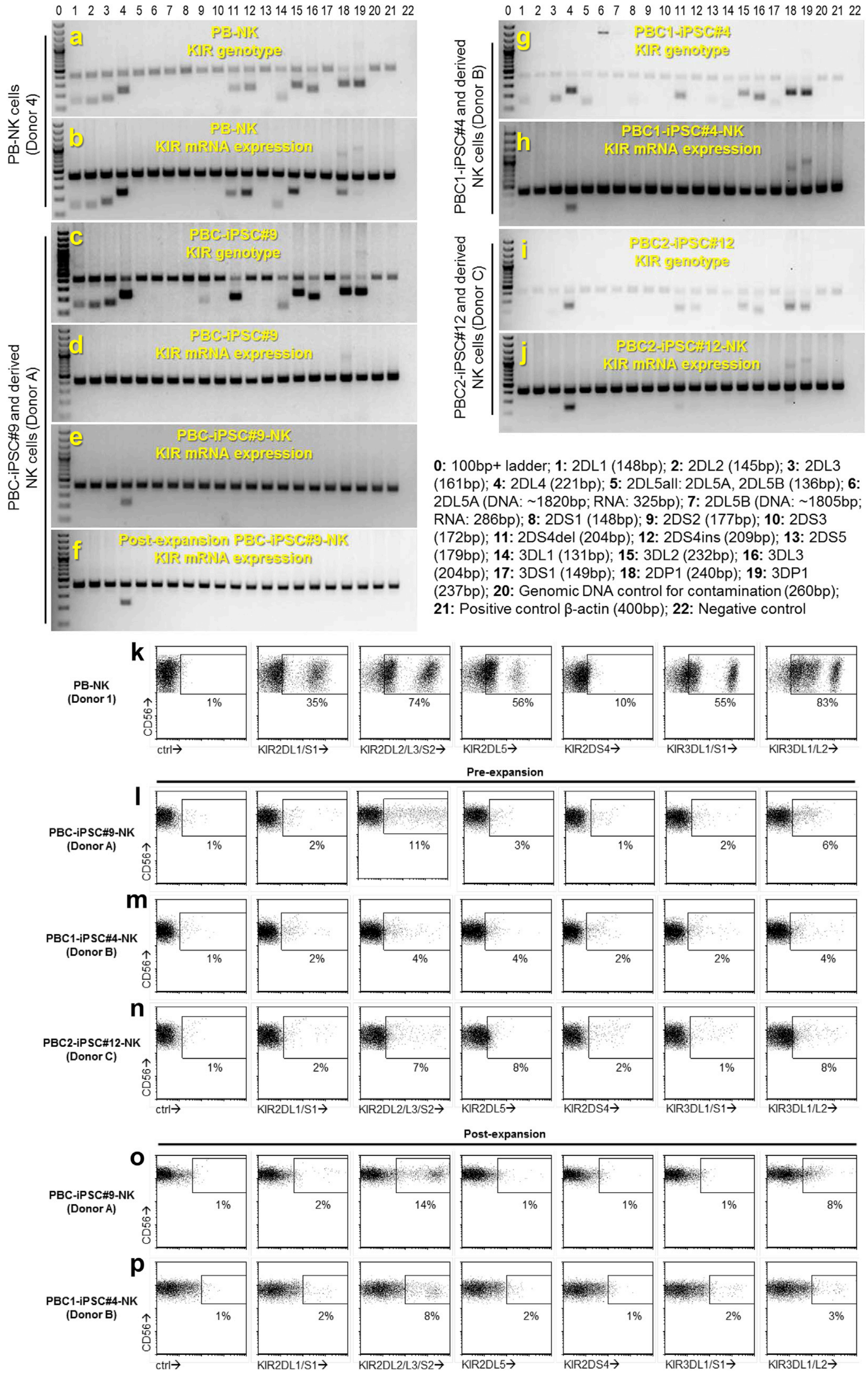

(legend on next page)

(2) our differentiation approach, which excludes cell sorting, EB formation, and spin EB formation in the process, and includes the use of OP9-DLL1 cells to provide active Notch signaling to induce lymphoid commitment, is more robust and practical; (3) the produced PBC-iPSC-NK cells are a high-purity and functional population, obviating the need for T and B cell depletion or NK cell enrichment; (4) both fresh and cryopreserved PBC-iPSC-NK cells can be expanded in a short period of time, which eases the logistics of manufacturing and transporting of these products; (5) further functional maturation and clinical-scale production can be achieved by cell expansion (starting with 3×10^6 PBC-iPSCs, 15×10^6 NK cells can be generated in 47 days; using feeder cells, these NK cells can be further expanded by 74-fold in 9–14 days; thus, by combining the differentiation and expansion processes, a total number of 1.1×10^9 potent NK cells can be produced to meet the clinical requirements not only in quantity but also in quality); and (6) most PBC-iPSC-NK cells are KIR negative, which may serve as a universal "off-the-shelf" cell source for various recipients. Interestingly, starting with an autologous PBC-iPSC line, it is possible to generate autologous KIR-negative NK cell source to be used under an autologous setting without worrying about the inhibition imposed by self HLA molecules. With a reduced risk of immune rejection, these autologous PBC-iPSC-NK cells may survive longer and thus provide a prolonged anti-tumor activity. However, it is noteworthy that the presented system relies on two stromal cell lines to differentiate hPSCs into NK cells that possess the above-mentioned unique product features. These cell lines could be the sources of cost and logistic problems during manufacturing. To further simplify the production process, the development of chemically defined coating matrix proteins that competently replace these cell lines could be certainly helpful.

In summary, we have established a "from blood cells to stem cells and back with less" strategy to mass-produce "off-the-shelf" NK cells from PBC-iPSCs for a wide range of recipients.

EXPERIMENTAL PROCEDURES

Generation of NK Cells from hPSCs

To generate NK cells from hPSCs, we established a two-stage protocol. In the first stage, OP9 cells were seeded on 0.1% gelatin (STEMCELL Technologies)-coated T75 flasks. Upon confluence, the cultures were fed by changing half of the medium and overgrown for 4–6 days. A total of 1–1.5 $\times 10^6$ hPSCs were then seeded and differentiated on the overgrown OP9 cells in alpha minimum essential medium (α-MEM) supplemented with 20% fetal bovine serum (FBS) for 12 days. The hPSC/OP9 co-cultures were fed every 4 days by changing half of the medium. In the second stage, the differentiated cells were harvested from the hPSC/OP9 co-cultures using 1 mg/mL Collagenase Type IV (STEMCELL Technologies) and TrypLE Express (Thermo Fisher Scientific). OP9 cells were removed by plastic adherence for 45 min and the cell clumps were further removed by 100 μm cell strainers (BD Biosciences). The remaining non-adherent cells were then co-cultured with OP9-DLL1 cells grown on T75 flasks using α-MEM containing 20% FBS, 10 ng/mL SCF (PeproTech), 5 ng/mL FLT3L (PeproTech) together with 5 ng/mL IL-7 (PeproTech), and/or 10 ng/mL IL-15 (PeproTech) for 7 days. Hereafter, the differentiated cells were harvested using Versene (Thermo Fisher Scientific) and co-cultured on new OP9-DLL1 cells grown on six-well plates on a weekly basis for another 3–4 weeks. After the final co-culture, the harvested cells were further purified by density gradient centrifugation using Ficoll-Paque PLUS (GE Healthcare Life Sciences) followed by overnight culture.

Generation of PBC-iPSCs

To generate iPSCs from PBCs, two different reprogramming protocols were established: one was using Sendai viral vectors (Thermo Fisher Scientific), while the other was using episomal reprogramming vectors (Thermo Fisher Scientific) as described in the Supplemental Experimental Procedures.

SUPPLEMENTAL INFORMATION

Supplemental Information includes Supplemental Experimental Procedures, two figures, and three figures and can be found with this article online at https://doi.org/10.1016/j.stemcr.2017.10.020.

AUTHOR CONTRIBUTIONS

J.Z., conception and design, collection and/or assembly of data, data analysis and interpretation, manuscript writing. S.Y.T., collection and/or assembly of data. L.L.T., collection and/or assembly of data. S.W., conception and design, data analysis and interpretation, manuscript writing, final approval of manuscript.

ACKNOWLEDGMENTS

This work was supported by the Singapore Ministry of Health's National Medical Research Council (NMRC/CIRG/1367/2013;

Figure 7. KIR Typing of PBC-iPSC-NK Cells

(A–J) KIR genotyping and mRNA expression profiling. Electrophoresis of PCR products of genomic DNA shows KIR gene content and endogenous β-actin gene of a PB-NK donor (donor 4) (A) and three PBC donors for PBC-iPSC#9 (donor A) (C), PBC1-iPSC#4 (donor B) (G), and PBC2-iPSC#12 (donor C) (I). Electrophoresis of PCR products of cDNA shows mRNA expression of KIR genes and endogenous β-actin gene in PB-NK cells (B), PBC-iPSC#9 cells (D), PBC-iPSC#9-NK cells (E), post-expansion PBC-iPSC#9-NK cells (F), PBC1-iPSC#4-NK cells (H), and PBC2-iPSC#12-NK cells (J).
(K–P) KIR phenotyping of different NK cells by flow cytometry.

NMRC/CIRG/1406/2014) and the Institute of Bioengineering and Nanotechnology (Biomedical Research Council, Agency for Science, Technology and Research, Singapore). The authors thank other lab members for helpful discussion and support.

Received: February 23, 2017
Revised: October 25, 2017
Accepted: October 25, 2017
Published: November 22, 2017

REFERENCES

Benson, D.M., Jr., and Caligiuri, M.A. (2014). Killer immunoglobulin-like receptors and tumor immunity. Cancer Immunol. Res. *2*, 99–104.

Benson, D.M., Jr., Hofmeister, C.C., Padmanabhan, S., Suvannasankha, A., Jagannath, S., Abonour, R., Bakan, C., Andre, P., Efebera, Y., Tiollier, J., et al. (2012). A phase 1 trial of the anti-KIR antibody IPH2101 in patients with relapsed/refractory multiple myeloma. Blood *120*, 4324–4333.

Benson, D.M., Jr., Cohen, A.D., Jagannath, S., Munshi, N.C., Spitzer, G., Hofmeister, C.C., Efebera, Y.A., Andre, P., Zerbib, R., and Caligiuri, M.A. (2015). A phase I trial of the anti-KIR antibody IPH2101 and lenalidomide in patients with relapsed/refractory multiple myeloma. Clin. Cancer Res. *21*, 4055–4061.

Colucci, F., Caligiuri, M.A., and Di Santo, J.P. (2003). What does it take to make a natural killer? Nat. Rev. Immunol. *3*, 413–425.

De Obaldia, M.E., and Bhandoola, A. (2015). Transcriptional regulation of innate and adaptive lymphocyte lineages. Annu. Rev. Immunol. *33*, 607–642.

Domogala, A., Madrigal, J.A., and Saudemont, A. (2015). Natural killer cell immunotherapy: from bench to bedside. Front. Immunol. *6*, 264.

Fujisaki, H., Kakuda, H., Shimasaki, N., Imai, C., Ma, J., Lockey, T., Eldridge, P., Leung, W.H., and Campana, D. (2009). Expansion of highly cytotoxic human natural killer cells for cancer cell therapy. Cancer Res. *69*, 4010–4017.

Gonzalez, F., Boue, S., and Izpisua Belmonte, J.C. (2011). Methods for making induced pluripotent stem cells: reprogramming a la carte. Nat. Rev. Genet. *12*, 231–242.

Jaleco, A.C., Neves, H., Hooijberg, E., Gameiro, P., Clode, N., Haury, M., Henrique, D., and Parreira, L. (2001). Differential effects of Notch ligands Delta-1 and Jagged-1 in human lymphoid differentiation. J. Exp. Med. *194*, 991–1002.

Karanu, F.N., Murdoch, B., Miyabayashi, T., Ohno, M., Koremoto, M., Gallacher, L., Wu, D., Itoh, A., Sakano, S., and Bhatia, M. (2001). Human homologues of Delta-1 and Delta-4 function as mitogenic regulators of primitive human hematopoietic cells. Blood *97*, 1960–1967.

Knorr, D.A., Ni, Z., Hermanson, D., Hexum, M.K., Bendzick, L., Cooper, L.J., Lee, D.A., and Kaufman, D.S. (2013). Clinical-scale derivation of natural killer cells from human pluripotent stem cells for cancer therapy. Stem Cells Transl. Med. *2*, 274–283.

Koehl, U., Brehm, C., Huenecke, S., Zimmermann, S.Y., Kloess, S., Bremm, M., Ullrich, E., Soerensen, J., Quaiser, A., Erben, S., et al. (2013). Clinical grade purification and expansion of NK cell products for an optimized manufacturing protocol. Front. Oncol. *3*, 118.

Lancaster, M.A., Renner, M., Martin, C.A., Wenzel, D., Bicknell, L.S., Hurles, M.E., Homfray, T., Penninger, J.M., Jackson, A.P., and Knoblich, J.A. (2013). Cerebral organoids model human brain development and microcephaly. Nature *501*, 373–379.

Lapteva, N., Szmania, S.M., van Rhee, F., and Rooney, C.M. (2014). Clinical grade purification and expansion of natural killer cells. Crit. Rev. Oncog. *19*, 121–132.

Leung, W. (2014). Infusions of allogeneic natural killer cells as cancer therapy. Clin. Cancer Res. *20*, 3390–3400.

Lim, O., Jung, M.Y., Hwang, Y.K., and Shin, E.C. (2015). Present and future of allogeneic natural killer cell therapy. Front. Immunol. *6*, 286.

McCullar, V., Oostendorp, R., Panoskaltsis-Mortari, A., Yun, G., Lutz, C.T., Wagner, J.E., and Miller, J.S. (2008). Mouse fetal and embryonic liver cells differentiate human umbilical cord blood progenitors into CD56-negative natural killer cell precursors in the absence of interleukin-15. Exp. Hematol. *36*, 598–608.

Miller, J.S. (2013). Therapeutic applications: natural killer cells in the clinic. Hematol. Am. Soc. Hematol. Educ. Program *2013*, 247–253.

Moretta, L., Pietra, G., Montaldo, E., Vacca, P., Pende, D., Falco, M., Del Zotto, G., Locatelli, F., Moretta, A., and Mingari, M.C. (2014). Human NK cells: from surface receptors to the therapy of leukemias and solid tumors. Front. Immunol. *5*, 87.

Murphy, W.J., Parham, P., and Miller, J.S. (2012). NK cells – from bench to clinic. Biol. Blood Marrow Transpl. *18*, S2–S7.

Nakano, T., Ando, S., Takata, N., Kawada, M., Muguruma, K., Sekiguchi, K., Saito, K., Yonemura, S., Eiraku, M., and Sasai, Y. (2012). Self-formation of optic cups and storable stratified neural retina from human ESCs. Cell Stem Cell *10*, 771–785.

Parham, P. (2005). MHC class I molecules and KIRs in human history, health and survival. Nat. Rev. Immunol. *5*, 201–214.

Tabatabaei-Zavareh, N., Vlasova, A., Greenwood, C.P., and Takei, F. (2007). Characterization of developmental pathway of natural killer cells from embryonic stem cells in vitro. PLoS One *2*, e232.

Thielens, A., Vivier, E., and Romagne, F. (2012). NK cell MHC class I specific receptors (KIR): from biology to clinical intervention. Curr. Opin. Immunol. *24*, 239–245.

Vey, N., Bourhis, J.H., Boissel, N., Bordessoule, D., Prebet, T., Charbonnier, A., Etienne, A., Andre, P., Romagne, F., Benson, D., et al. (2012). A phase 1 trial of the anti-inhibitory KIR mAb IPH2101 for AML in complete remission. Blood *120*, 4317–4323.

Vodyanik, M.A., Bork, J.A., Thomson, J.A., and Slukvin, I.I. (2005). Human embryonic stem cell-derived CD34+ cells: efficient production in the coculture with OP9 stromal cells and analysis of lymphohematopoietic potential. Blood *105*, 617–626.

Woll, P.S., Martin, C.H., Miller, J.S., and Kaufman, D.S. (2005). Human embryonic stem cell-derived NK cells acquire functional receptors and cytolytic activity. J. Immunol. *175*, 5095–5103.

Woll, P.S., Grzywacz, B., Tian, X., Marcus, R.K., Knorr, D.A., Verneris, M.R., and Kaufman, D.S. (2009). Human embryonic stem cells differentiate into a homogeneous population of

natural killer cells with potent in vivo antitumor activity. Blood *113*, 6094–6101.

Yang, J., Lam, D.H., Goh, S.S., Lee, E.X., Zhao, Y., Tay, F.C., Chen, C., Du, S., Balasundaram, G., Shahbazi, M., et al. (2012). Tumor tropism of intravenously injected human-induced pluripotent stem cell-derived neural stem cells and their gene therapy application in a metastatic breast cancer model. Stem Cells *30*, 1021–1029.

Zeng, J., Shahbazi, M., Wu, C., Toh, H.C., and Wang, S. (2012). Enhancing immunostimulatory function of human embryonic stem cell-derived dendritic cells by CD1d overexpression. J. Immunol. *188*, 4297–4304.

Zeng, J., and Wang, S. (2014). Human dendritic cells derived from embryonic stem cells stably modified with CD1d efficiently stimulate antitumor invariant natural killer T cell response. Stem Cells Transl. Med. *3*, 69–80.

Zeng, J., Wu, C., and Wang, S. (2015). Antigenically modified human pluripotent stem cells generate antigen-presenting dendritic cells. Sci. Rep. *5*, 15262.

Stem Cell Reports, Volume *9*

Supplemental Information

Generation of “Off-the-Shelf” Natural Killer Cells from Peripheral Blood Cell-Derived Induced Pluripotent Stem Cells

Jieming Zeng, Shin Yi Tang, Lai Ling Toh, and Shu Wang

Supplemental Information

Generation of "Off-the-Shelf" Natural Killer Cells from Peripheral Blood Cell-derived Induced Pluripotent Stem Cells

Jieming Zeng[1], Shin Yi Tang[1,2], Lai Ling Toh[1] and Shu Wang[1,2]

[1]Institute of Bioengineering and Nanotechnology, Singapore
[2]Department of Biological Sciences, National University of Singapore, Singapore

Inventory of Supplemental Information

1. Supplemental Data

Figure S1, related to Figure 3c;

Figure S2, related to Figure 7;

Table S1, related to Figure 3;

Table S2, related to Figure 7;

Table S3, related to discussion.

2. Supplemental Experimental Procedures

3. References

Supplemental Experimental Procedures

Cell culture. A hESC line, H1 (WiCell Research Institute, Madison, WI, http://www.wicell.org) and a previously generated fibroblast-derived iPSC line, iPSC#5.9[1], were cultured with mTeSR1 (StemCell Technologies, Vancouver, BC, Canada, http://www.stemcell.com) on Matrigel (BD Biosciences, San Diego, CA, http://www.bdbiosciences.com) -coated six-well plates. Cell lines: OP9, K562, Raji, SK-OV-3, SW480, HCT-8, MCF-7 and SCC-25 (American Type Culture Collection [ATTC], Manassas, VA, http://www.atcc.org) were cultured as recommended by ATCC. Cell line OP9-DLL1 (Riken BRC Cell Bank, Ibaraki, Japan, http://cell.brc.riken.jp/en/) was cultured in α-MEM (Thermo Fisher Scientific, Waltham, MA, http://corporate.thermofisher.com) supplemented with 20% fetal bovine serum (FBS) (HyClone, Logan, UT, http://www.hyclone.com). Cell line K562-mbIL15-41BBL (kindly provided by Dr. D. Campana, Yong Loo Lin School of Medicine, National University of Singapore) was cultured in IMDM (Thermo Fisher Scientific) supplemented with 10% FBS.

Generation of PBC-iPSCs. To generate iPSCs from PBCs, frozen PBMCs from a healthy donor (StemCell Technologies) were thawed and cultured with 5 μg/ml phytohemagglutinin (PHA; Sigma-Aldrich, St Louis, MO, http://www.sigmaaldrich.com) for 2 days in complete RPMI 1640 medium, which is composed of RPMI 1640, 10% heat-inactivated human serum AB (Gemini Bio-Products, West Sacramento, CA, http://www.gembio.com), 2 mM L-glutamine (Thermo Fisher Scientific) and 0.1 mM nonessential amino acids (Thermo Fisher Scientific). The cultured blood cells were then transduced with Sendai reprogramming vectors from a CytoTune iPS 2.0 Sendai Reprogramming Kit (Thermo Fisher Scientific) at MOI of 5:5:3 (KOS, hc-Myc, hKlf4) in complete RPMI 1640 medium containing 10 ng/ml of IL-2, IL-7 and IL-15 overnight. The transduced cells were then washed and cultured for 5 days before seeding to a six-well plate grown with mitomycin C (Sigma-Aldrich) -treated mouse embryonic fibroblasts (mEFs). Half of medium was replaced on day 1 to 3 after seeding with iPSC medium, which is composed of DMEM/F12 (Thermo Fisher Scientific), 20% knockout serum replacement (Thermo Fisher Scientific), 2 mM L-glutamine, 1% nonessential amino acids, 0.1 mM 2-mercaptoethanol and 5 ng/ml basic fibroblast growth factor (PeproTech). Three to four weeks after seeding, the resulting iPSCs were first expanded on mEFs in iPSC medium and later on Matrigel-coated plates in mTeSR1.

To generate non-T cell-derived PBC-iPSCs, PBMCs from healthy donors were cultured in CD34+ cells enrichment medium, which is composed of StemPro-34 medium (Thermo Fisher Scientific), 100 ng/ml SCF, 50 ng/ml IL-3 (PeproTech) and 25 ng/ml GM-CSF (PeproTech) for three days. At day 0, episomal reprogramming vectors from a Epi5 Episomal iPSC Reprogramming Kit (Thermo Fisher Scientific) were delivered into the cultured PBMCs via nucleofection using a Amaxa Nucleofector 2b (Lonza, http://www.lonza.com). The nuleofected cells were then seeded on mitomycin C-inactivated mEFs in CD34+ cell enrichment medium. On day 2, the cells were adapted to a 1:1 mixture of CD34+ cells enrichment medium: iPSC medium. From day 3 on, the cells were cultured in iPSC medium, which was changed every other day. Two to four weeks after seeding, iPSC colonies were picked and expanded in Matrigel-coated six-well plates in mTeSR1.

TCRB and TCRG gene clonality assays. Genomic DNA was isolated from PBC-iPSCs using a DNeasy Blood and Tissue Kit (Qiagen, https://www.qiagen.com) according to the manufacturer's instruction. To detect TCRβ and TCRγ gene rearrangement in genomic DNA, PCR was carried out with master mixes provided in TCRB and TCRG Gene Clonality Assay kits (Invivoscribe Technologies, San Diego, CA, http://www.invivoscribe.com) and AmpliTaq Gold DNA polymerase (Thermo Fisher Scientific) using the following program: 95ºC for 7 minutes; 35 amplification cycles (95ºC for 45 seconds, 60ºC for 45 seconds, 72ºC for 90 seconds); and final extension of 72ºC for 10 minutes before holding at 15ºC. PCR products were separated by electrophoresis in 2% MetaPhor Agarose (Lonza, http://www.lonza.com) gel.

Flow cytometry. To study phenotypic change during hPSC differentiation, cells were harvested and stained using antibodies against CD34 (BD Biosciences, cat#550761 and cat#555824), CD43 (BD Biosciences, cat#555475), CD45 (BD Biosciences, cat#555483 and cat#557748), CD56 (BD Biosciences, cat#555518), TCRαβ (BD Biosciences, cat#564728), CD3 (BD Biosciences, cat#555333 and cat#561811), CD4 (BD Biosciences, cat#557852), CD8 (BD Biosciences, cat#555634), CD19 (BD Biosciences, cat#561741), CD14 (Thermo Fisher Scientific, cat#12-0149-42), NKp30 (BD Biosciences, cat#558407), NKp44 (BD Biosciences, cat#558563), NKp46 (BD Biosciences, cat#557991), NKG2D (BD Biosciences, cat#557940), NKG2A (Beckman Coulter, cat#IM3291U), CD94 (Thermo Fisher Scientific, cat#12-0949-42), CD16 (BD Biosciences, cat#560995), FasL (BD Biosciences, cat#564261), TRAIL (BD Biosciences, cat#561784), DNAM-1 (BD Biosciences, cat#559789), CD158a,h (KIR2DL1/S1) (Beckman Coulter, cat#A09778), CD158b (KIR2DL2/L3/S2) (Miltenyi Biotec, cat#130-092-618), CD158f (KIR2DL5) (Miltenyi Biotec, cat#130-096-199), CD158i (KIR2DS4) (Miltenyi Biotec, cat#130-092-680), CD158e1/e2 (KIR3DL1/S1) (Beckman Coulter,

cat#IM3292) and CD158e/k (KIR3DL1/L2) (Miltenyi Biotec, cat#130-095-205) and analyzed with a FACSCalibur flow cytometer (BD Biosciences). Isotype controls were used accordingly for flow cytometry.

ELISPOT assay. To detect IFN-γ secretion, a Human IFN-γ ELISpotPro kit (MABTECH, Nacka Strand, Sweden, https://www.mabtech.com) was used. In brief, 0 to 10×10^4 PBC-iPSC-NK cells and 5×10^4 K562 or Raji cells were cocultured on a IFN-γ ELISPOT plate overnight. IFN-γ spots were stained according to the manufacturer's manual. To measure GrB secretion, a Human Granzyme B ELISpot Kit (R&D Systems, Minneapolis, MN, https://www.rndsystems.com) was used. In brief, 0 to 1.2×10^4 PBC-iPSC-NK cells were incubated with or without 5×10^4 K562 cells on a human GrB microplate for 4 hours. GrB spots were then stained as described in the manufacturer's manual. IFN-γ and GrB spots were counted using an ImmunoSpot Analyzer (CTL, Shaker Heights, OH, http://www.immunospot.com).

Cytotoxicity and ADCC assay. To detect direct cytotoxicity of PBC-iPSC-NK cells against target cells, a flow cytometry-based method was used. In brief, 0 to 0.5×10^6 PBC-iPSC-NK cells were cocultured with 2×10^4 carboxyfluorescein diacetate succinimidyl ester (CFSE; Thermo Fisher Scientific) -labelled cancer cells at various effector to target (E:T) ratios for 4-6 hours. Samples were then stained on ice with 7-Amino-Actinomycin D (7-AAD, BD Biosciences) for 10 minutes. After washing, target cell death was assessed with flow cytometer by the percentage of 7-AAD-stained cells in CFSE-positive population. To evaluate ADCC function of PBC-iPSC-NK cells, cocultures of NK cells and CFSE-labelled Raji cells were set up at the indicated E:T ratios in the presence of human IgG1 (Sigma-Aldrich), anti-CD20-mIgG1 (InvivoGen, San Diego, CA, http://www.invivogen.com) or anti-CD20-hIgG1 (InvivoGen) of various concentrations, Raji cell death was measured after 4-hour incubation by flow cytometry as described above.

Expansion of fresh and cryopreserved PBC-iPSC-NK cells. To expand fresh PBC-iPSC-NK cells, 0.25-1×10^6 purified NK cells were cocultured with γ- irradiated (100 Gy) K562-mbIL15-41BBL cells at an NK cell: feeder cell ratio of 1:10 in gas-permeable G-Rex10 flasks (Wilson Wolf Manufacturing, New Brighton, MN, http://www.wilsonwolf.com) in 40 ml CellGro SCGM serum-free medium (Cell Genix, Freiburg, Germany, http://www.cellgenix.com) supplemented with 10% FBS, 20 IU/ml IL-2 (PeproTech), 10 ng/ml SCF, 5 ng/ml FLT3L, 5 ng/ml IL-7 and 10 ng/ml IL-15. Fresh cytokines were replenished every 2-3 days and 30 ml medium was replaced with fresh medium every 5 days during the 14-day expansion. Numbers of NK cells were counted at every medium change. To cryopreserve PBC-iPSC-NK cells, NK cells were resuspended in cold CryoStor CS10 (BioLife Solutions, Bothell, WA, http://www.biolifesolutions.com/) and frozen at -80°C in a Mr. Frosty (Thermo Fisher Scientific) overnight before storing in liquid nitrogen. To study the expansion of cryopreserved PBC-iPSC-NK cells, the NK cells were thawed and cell viability and live cell number were determined before and after coculture with K562-mbIL15-41BBL.

Expansion of donor-derived PB-NK cells. To derive PB-NK cells, 2×10^6 PBMCs from healthy donors were cocultured with 4×10^6 γ-irradiated (100 Gy) K562-mbIL15-41BBL cells in CellGro SCGM serum-free medium supplemented with 10% FBS and 50 IU/mL IL-2 using T75 flasks in upright position. Half of the medium was replaced with fresh medium and fresh IL-2 was replenished every 2-3 days until day 7. Hereafter, 2×10^6 cells were re-stimulated weekly with 2×10^6 K562-mbIL15-41BBL cells for another two weeks. The cells were harvested on day 21 for experiments.

Derivation of primary tumor cells. To test the cytotoxicity of NK cells against primary tumor cells, short-term cultures of primary tumor cells were established from fresh colorectal cancer samples using a protocol described previously[2]. The samples were obtained from patients under treatment at National Cancer Centre Singapore and the study was approved by local Institutional Review Board. In brief, the tumor samples were finely minced and further dissociated into very small tissue fragments by vigorous pipetting. The tissue fragments were then resuspended in AR-5 initiation medium [2] and grown in cell culture dishes to establish primary cultures. Upon heavy growth of tumor cells, the adherent colonies were scraped off under a stereomicroscope for further culture. Such derived pure cancer cells were then propagated in RPMI 1640 medium supplemented with 10% FBS. These cancer cells were characterized by immunostaining using antibodies against cytokeratin (Miltenyi Biotec, cat#130-080-101), vimentin (BD Biosciences, cat#562337), CD133 (BD Biosciences, cat#566593), CK20 (Santa Cruz, cat#sc-271183) and flow cytometry using antibodies against CD133, CD44 (Miltenyi Biotec, cat#130-095-177) and EpCAM (Miltenyi Biotec, cat#130-091-254) and then used as target cells in cytotoxicity assay between passage 10 to 12.

KIR typing. To detect KIR genes on genomic DNA level and to analyze of KIR expression on mRNA level, a KIR typing kit (Miltenyi Biotec) was used according to manufacturer's instruction.

References

1. Yang, J. et al. Tumor tropism of intravenously injected human-induced pluripotent stem cell-derived neural stem cells and their gene therapy application in a metastatic breast cancer model. *Stem cells* **30**, 1021-1029 (2012).
2. Park, J.G., Ku, J.L. & Park, S.Y. Isolation and culture of colon cancer cell lines. *Methods in molecular medicine* **88**, 79-92 (2004).

Figure S1

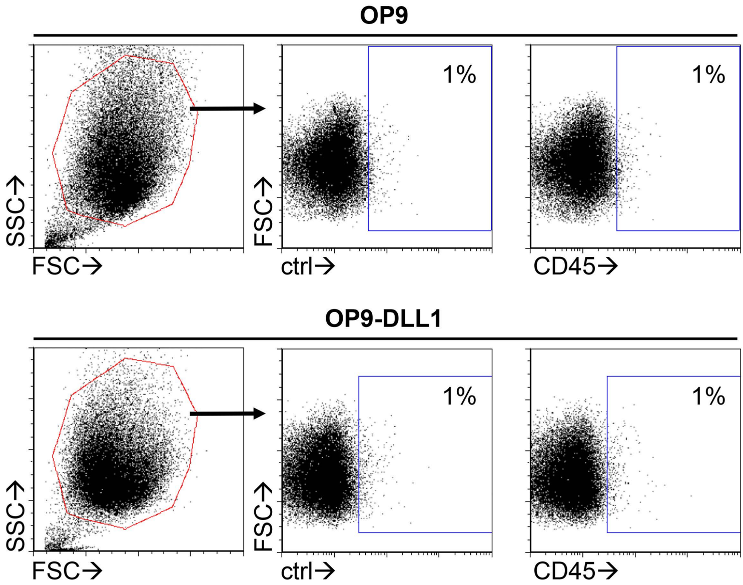

Fig. S1: CD45 expression in OP9 and OP9-DLL1. OP9 and OP9-DLL1 cells were stained by an anti-human CD45 monoclonal antibody and its isotype control. Flow cytometric analysis showed no CD45 expression in both OP9 and OP9-DLL1.

Figure S2

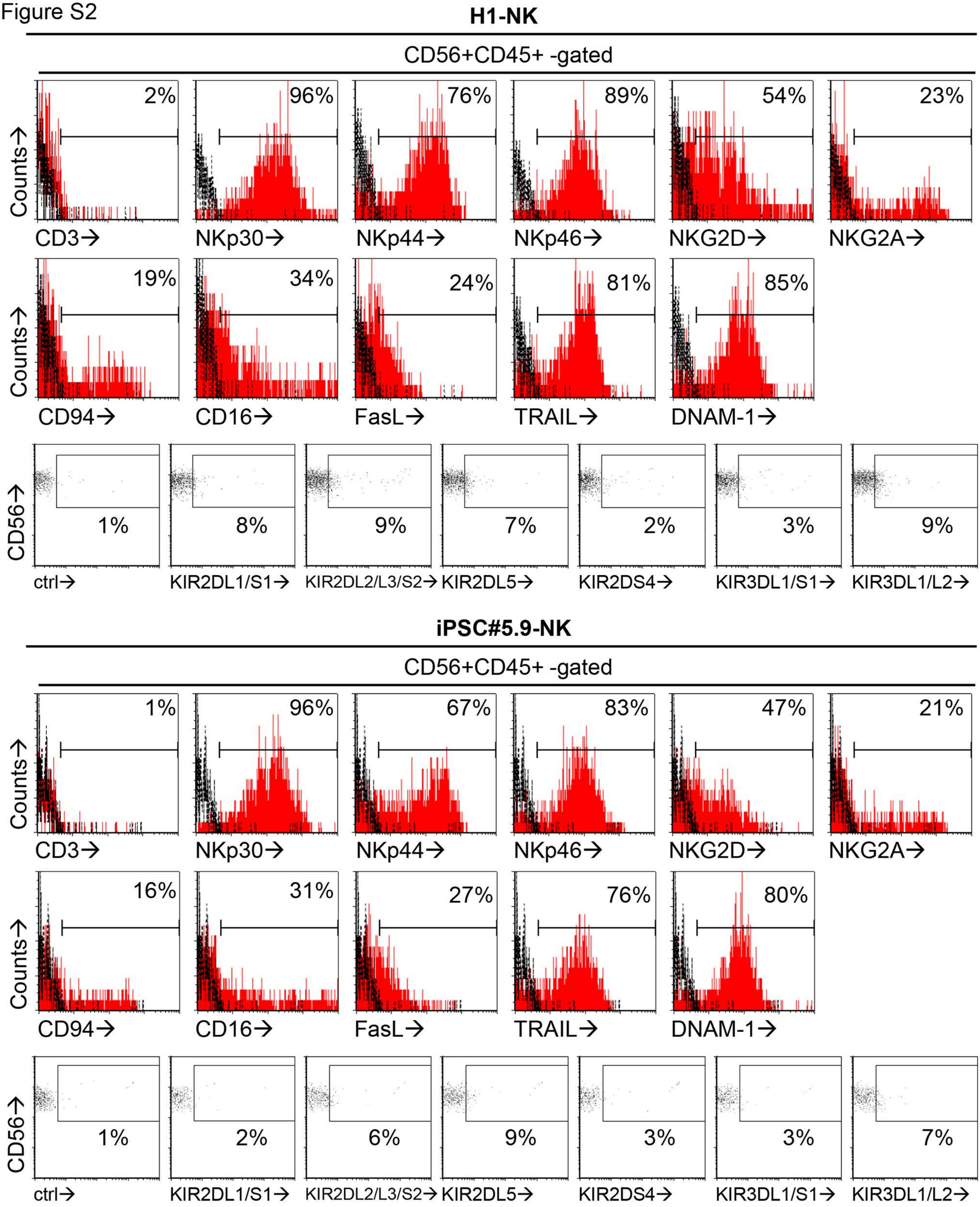

Fig. S2: Phenotype of NK cells generated from hESCs and fibroblast-derived iPSCs. A hESC line, H1 and a fibroblast-derived iPSC line, iPSC#5.9 were used to generate NK cells.

Table S1: A Cytokine Cocktail Improved Purity and Yield of PBC-iPSC-NK Cells

<table>
<tr><th>Cytokines</th><th>SCF, FLT3L
+ IL-7</th><th>SCF, FLT3L
+ IL-15</th><th>SCF, FLT3L
+ IL-7, IL-15</th></tr>
<tr><td>% of
CD56+
CD45+
(purity)</td><td>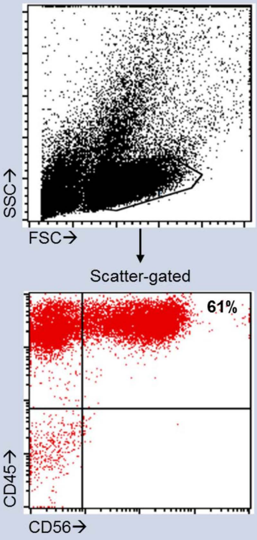
</td><td>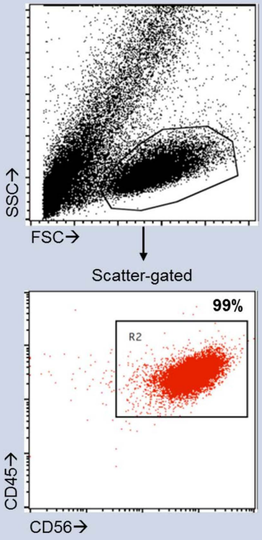
</td><td>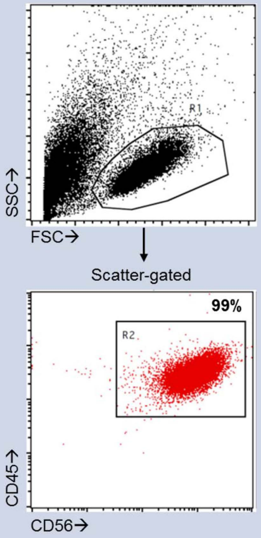
</td></tr>
<tr><td># of
CD56+
CD45+
(yield)</td><td>$0.21 \times 10^6 \pm 0.18 \times 10^6$
per 3×10^6 iPSCs
(mean ± SD, n=4, d40)</td><td>$0.75 \times 10^6 \pm 0.43 \times 10^6$
per 3×10^6 iPSCs
(mean ± SD, n=3, d40)</td><td>$7.93 \times 10^6 \pm 0.18 \times 10^6$
per 3×10^6 iPSCs
(mean ± SD, n=6, d40)</td></tr>
</table>

Table S2: KIR Genotype and mRNA Expression of PBC-iPSC-NK Cells

KIR gene		2DL1	2DL2	2DL3	2DL4	2DL5all	2DL5A	2DL5B	2DS1	2DS2	2DS3	2DS4del	2DS4ins	2DS5	3DL1	3DL2	3DL3	3DS1	2DP1	3DP1
PB-NK (Donor 4)	KIR genotype	+	+	+	+	-	-	-	-	-	-	+	+	-	+	+	+	-	+	+
	KIR mRNA expression	+	+	+	+	-	-	-	-	-	-	+	+	-	+	+	-	-	+	+
PBC-iPSC#9-NK (Donor A)	KIR genotype	+	+	+	+	-	-	-	-	+	-	+	-	-	+	+	+	-	+	+
	KIR mRNA expression	-	-	-	+	-	-	-	-	-	-	-	-	-	-	-	-	-	-	-
PBC1-iPSC#4-NK (Donor B)	KIR genotype	+	-	+	+	+	+	+	+	-	-	+	-	+	+	+	+	+	+	+
	KIR mRNA expression	-	-	-	+	-	-	-	-	-	-	-	-	-	-	-	-	-	-	-
PBC2-iPSC#12-NK (Donor C)	KIR genotype	-	-	+	+	-	-	-	-	-	-	+	+	-	-	+	+	-	+	+
	KIR mRNA expression	-	-	-	+	-	-	-	-	-	-	+	+	-	-	-	-	-	+	-

Table S3: Comparison of Previous and Current Technologies for Production of NK Cells from hPSCs					
**Color Code**		**Pro**	**Con**		**Neutral**
		**Previous Technology Described by Knorr et al, 2013**			**Current Technology Presented by Zeng et al, 2017**
**Protocols**		**Protocol A - Feeder-dependent protocol #1:** stromal cells M210-B4 for stage I; stromal cells EL08-1D2 for stage II.	**Protocol B - Feeder-dependent protocol #2:** "spin EBs" for stage I; stromal cells EL08-1D2 for stage II; expansion with aAPCs for stage III.	**Protocol C - Feeder-free protocol:** "spin EBs" for stage I; feeder-free method for stage II; expansion with aAPCs for stage III.	**Protocol D - Feeder-dependent protocol:** stromal cells OP9 for stage I; stromal cells OP9-DLL1 for stage II; expansion with aAPCs for stage III.
**Starting hPSCs**	**hESCs**	H1, H9	H9	H9	H1
	**Fibroblast-derived iPSCs**	BJ1-iPSC, DRiPS16	not tested	not tested	iPSC#5.9
	**UCB-derived iPSCs**	UCBiPS7	UCBiPS7	not tested	not tested
	**PBC-derived iPSCs**	not tested	not tested	not tested	PBC-iPSC#9, PBC1-iPSC#4, PBC2-iPSC#12 **[Comment: Generation of GMP-ready iPSCs from PBCs is "a rountine procedure that is not novel". However, the feasibility to use these PBC-derived GMP-ready iPSCs to generate NK cells has never been demonstrated previously.]**
**Stage I: Method for differentiating hPSCs to precursor cells**	**Cell processing**	**Feeder-dependent method:** Coculture hPSCs with stromal cells M210-B4. **[Comment: "the use of murine stromal layers does not absolutely prohibit clinical translation if master cells banks are used." - a direct quote from Knorr et al, 2013]**	**"Spin EB" method:** **Step 1 - Adaptation of hPSCs to TrypLE:** Passage hPSCs using TrypLE on mEFs (mouse embryonic fibroblasts) for at least 10 passages; **Step 2 - Spin EB formation:** Seed TrypLE-passaged hPSCs on 96-well plate at 3000 cells/well; spin to aggregate the cells; culture to form EBs. **[Comments: 1. mEFs are mouse cells. Thus, the described spin EB method is not a xeno-free process; 2. Spin EB formation is laborious.]**		**Feeder-dependent method:** Coculture hPSCs with stromal cells OP9. **[Comment: "the use of murine stromal layers does not absolutely prohibit clinical translation if master cells banks are used." - a direct quote from Knorr et al, 2013]**
	**Medium**	RPMI1640 + 15% FBS **[Comment: There are gamma-irradiated GMP-grade FBS from different manufacturers such as Lonza, Gibco and Hyclone, which can be used for GMP-complying cell culture.]**	**Step 1 - Adaptation of hPSCs to TrypLE:** Undescribed medium; **Step 2 - Spin EB formation:** BPEL medium + SCF, BMP-4 and VEGF. **[Comments: 1. Undescribed medium was used for adaptation; not sure if it is xeno-free; 2. BPEL (bovine serum albumin polyvinyl alchohol essential lipids) medium contains bovine serum albumin. Thus, the described spin EB method is not a xeno-free process.]**		αMEM + 20% FBS **[Comment: There are gamma-irradiated GMP-grade FBS from different manufacturers such as Lonza, Gibco and Hyclone, which can be used for GMP-complying cell culture.]**
	**Duration**	21 days	**Step 1 - Adaptation of hPSCs to TrypLE:** at least 10 passages (~10 weeks); **Step 2 - Spin EB formation:** 11 days. **[Comment: Knorr et al. have used a laborious TrypLE adaptation approach. This approach is not necessary for hPSC aggregation, since the inclusion of ROCK inhibitor in the medium is sufficient for any hPSC to form aggregates in U-bottom 96-well plates following TrypLE dissociation. However, it remains to be tested whether the pretreatment with ROCK inhibitor will affect the differentiation of hPSCs into NK cells.]**		12 days **[Comment: Protocol D has the shortest duration for stage I comparing with Protocol A, B and C.]**
	**Precursor cell processing**	Sorting CD34+CD45+ cells	No cell sorting required		No cell sorting required
**Stage II: Method for differentiating precursor cells to NK cells**	**Cell processing**	**Feeder-dependent method:** Coculture sorted CD34+CD45+ cells with stromal cells EL08-1D2. **[Comment: "the use of murine stromal layers does not absolutely prohibit clinical translation if master cells banks are used." - a direct quote from Knorr et al, 2013]**	**Feeder-dependent method:** Transfer 6 wells of spin EBs from 96-well plate into one well of 24-well plate pre-seeded with stromal cells EL08-1D2. **[Comment: It is laborious for scale-up production.]**	**Feeder-free method:** Transfer 6 wells of spin EBs from 96-well plate into one uncoated well of 24-well plate. **[Comment: It is laborious for scale-up production.]**	**Feeder-dependent method:** Coculture harvested differentiated cells with stromal cells OP9-DLL1. **[Comment: "the use of murine stromal layers does not absolutely prohibit clinical translation if master cells banks are used." - a direct quote from Knorr et al, 2013]**
	**Medium**	Unknown medium + NK cell initiaiting cytokines. **[Comment: Undescribed medium was used, not sure if it is xeno-free.]**			αMEM + 20% FBS + SCF, FLT3L, IL-7, IL-15 **[Comment: There are gamma-irradiated GMP-grade FBS from different manufacturers such as Lonza, Gibco and Hyclone, which can be used for GMP-complying cell culture.]**
	**Duration**	28-35 days			28-35 days
**hPSC-derived NK cells (before expansion)**	**Yield**	?, not described	~1e6 NK cells per 1.8e4 H9 cells (Fig. 3A). **[Comment: Only H9 cells were used to test Protocol B. It is not sure whether Protocol B is applicable to other hPSC lines/sources.]**	~0.72e6 NK cells per 1.8e4 H9 cells (Fig. 3A). **[Comment: Only H9 cells were used to test Protocol B. It is not sure whether Protocol B is applicable to other hPSC lines/sources.]**	15e6 NK cells per 3e6 PBC-iPSCs **[Comment: These NK cells can be further expanded by up to 74-fold and the number of expanded NK cells (1.1e9) is enough for clinical application.]**
	**Purity**	?, not described	?, not described **[Comment: As estimated from Fig. 2C (the FSC v CD56 dot plots for ES-derived NK cells +EL08 and UCBiPS-derived NK cells +EL08), the purities seemed reasonable.]**	?, not described. **[Comment: As estimated from Fig. 2C (FSC v CD56 dot plot of ES-derived NK cells -EL08), there was a substantial CD56-negative population. The derived population might not be high-purity.]**	up to 99%
	**Phenotype**	high-level KIR expression (Fig. 1C and Fig. 2C). **[Comment: Binding of KIR ligands to KIRs suppresses cytotoxicity of donor NK cells against patient's cancer cells. To improve clinical outcome, elaborately selecting an NK cell donor for a particular patient to obtain a KIR-HLA mismatch in an anti-cancer direction is critical. Besides using NK cells derived from a KIR-HLA mismatched donor, a blocking anti-KIR antibody has also been used in clinical trials to reduce inhibition of NK cells via KIR signaling. These suggest that NK cells with high-level KIR expression may only be applicable in a certain group of patients and high-level KIR expression is not a desirable feature for "off-the-shelf" application.]**			KIR-negative. **[Comment: A unique KIR-negative phenotype renders PBC-iPSC-NK cells unrestricted by reciepient's HLA genotypes and thus potent cytotoxicity against cancer cells. Since KIR-based therapeutic intervention is unnecessary, PBC-iPSC-NK cells may serve as a universal "off-the-shelf" cell source for many recipients.]**
	**Cytoxicity against NK-sensitive K562 cells (Potencies of NK cells generated using various protocols and PB-NK are compared.)**	NK cells generated from Protocol A only killed 37% of K562 cells at E:T ratio of 10 (supplemental Fig. 2). **[Comment: NK cells generated with Protocol A are not potent.]**	NK cells generated from Protocol B and Protocol C killed up to 75% of K562 at E:T ratio of 10 (Fig. 2D). **[Potency ranking: Protocol B ≈ Protocol C ≈ PB-NK.]**		NK cells generated from Protocol D killed up to 90% of K562 at E:T ratio of 10. **[Potency ranking: Protocol D > Protocol B ≈ Protocol C ≈ PB-NK > Protocol A]**
	**Cytotoxicity against multiple myeloma cell lines**	not tested	Potency ranking: PB-NK > Protocol B > Protocol C (Fig. 4B). **[Comment: NK cells generated from Protocol C have the worse potency among three tested NK cell populations. This is further supported by the IFN-γ and CD107a data (Fig. 4A).]**		not tested

	**Cytotoxicity against pancreatic cancer cell lines**	not tested	Potency ranking: PB-NK > Protocol B (Fig. 4B)	not tested	not tested
**Stage III: Method to expand hPSC-derived NK cells**	**Cell processing**	not tested	H9-derived NK cells were cocultured with 9.mbIL-21 aAPC with a aAPC:NK ratio of 2:1 on day 0 and with a ratio of 1:1 weekly thereafter until d70. **[Comments: 1. Laborious cell processing: There were 10 cocultures required during the 70-day process; 2. Requirement of a large and undefined number of aAPCs: Need to prepare aAPC every week for 10 weeks and the number of required aAPC will increase weekly with the expansion of NK cells; 3. High risk of contamination due to long-term cell culture.]**		PBC-iPSC-derived NK cells were cocultured with K562-mbIL15-41BBL with a aAPC:NK ratio of 10:1 on day 0. Expanded NK cells are harvested between day 9 -14. **[Comment: 1. Easy cell processing: Only need one coculture at the beginning; 2. Requirement of a small and defined number of aAPCs: Only need to prepare aAPCs once; 3. Low risk of contamination due to short-term cell culture.]**
	**Medium**	not tested	RPMI1640 + 15% FBS. **[Comment: There are gamma-irradiated GMP-grade FBS from different manufacturers such as Lonza, Gibco and Hyclone, which can be used for GMP-complying cell culture.]**		CellGro SCGM serum-free medium + 10% FBS. **[Comment: There are gamma-irradiated GMP-grade FBS from different manufacturers such as Lonza, Gibco and Hyclone, which can be used for GMP-complying cell culture.]**
	**Duration**	not tested	70 days		9 - 14 days
**Expanded hPSC-derived NK cells**	**Expansion fold**	not tested	100 to 1000-fold in 70 days		up to 74-fold in 14 days
	**Clinical-scale Production (From hPSCs --> NK cells -> expanded NK cells)**	not tested	0.25e6 H9 cells -- (x56.8) --> 14e6 NK cells -- (x100) --> 1.4e9 in 116 days. **[Comment: Clinical-scale production is achievable through long-term expansion.]**	0.25e6 H9 cells -- (x40.4) --> 10e6 NK cells -- (x100) --> 1e9 in 116 days. **[Comment: Clinical-scale production is achievable through long-term expansion.]**	3e6 PBC-iPSCs -- (x5) --> 15e6 NK cells -- (x74) --> 1.1e9 in 61 days. **[Comment: Clinical-scale production is achievable through short-term expansion.]**
	**Purity**	not tested	up to 99%		up to 99%
	**Phenotype**	not tested	high-level KIR expression (Fig. 5B). **[Comment: Binding of KIR ligands to KIRs suppresses cytotoxicity of donor NK cells against patient's cancer cells. To improve clinical outcome, elaborately selecting an NK cell donor for a particular patient to obtain a KIR-HLA mismatch in an anti-cancer direction is critical. Besides using NK cells derived from a KIR-HLA mismatched donor, a blocking anti-KIR antibody has also been used in clinical trials to reduce inhibition of NK cells via KIR signaling. These suggest that NK cells with high-level KIR expression may only be applicable in a certain group of patients and high-level KIR expression is not a desirable feature for "off-the-shelf" application.]**		KIR-negative. **[Comment: A unique KIR-negative phenotype renders PBC-iPSC-NK cells unrestricted by reciepient's HLA genotypes and thus potent cytotoxicity against cancer cells. Since KIR-based therapeutic intervention is unnecessory, PBC-iPSC-NK cells may serve as a universal "off-the-shelf" cell source for many recipients.]**
	**Cytotoxicity against NK-sensitive K562 cells**	not tested	15% killing (vs 62% killing before expansion) at E:T ratio of 2.5; 60% killing (vs 75% killing before expansion) at E:T ratio of 10 (Fig. 5D). **[Comment: This decrease in potency suggests that the NK cells are funtionally exhausted after prolonged expansion.]**		up to 65% killing (vs up to 35% killing before expansion) at E:T ratio of 2; up to 90% killing (vs up to 80% killing before expansion) at E:T ratio of 10. **[Comment: This increase in potency suggests that the NK cells are more funtionally mature after short-term expansion.]**
	**Cytotoxicity against other cancer cell lines**	not tested	not tested		effiently kill a wide range of solid tumor cell lines; more efficient than PB-NK (Fig. 6). **[Comment: The high potency of expanded NK cells is likely due to: (a) the KIR-negative phenotype and (b) the short-term expansion of NK cells.]**
